# Correction: Consensus structure prediction of A. thaliana’s MCTP4 structure using prediction tools and coars grained simulations of transmembrane domain dynamics

**DOI:** 10.1371/journal.pone.0335771

**Published:** 2025-10-30

**Authors:** Sujith Sritharan, Raphaelle Versini, Jules D Petit, Emmanuelle E Bayer, Antoine Taly

The Acknowledgment section for this article is missing. The correct Acknowledgment section is as follows: This work was granted access to the HPC resources of IDRIS under the allocation 2024-A0160715149 made by GENCI to AT.
