# Supplementary material for: Consensus structure prediction of A. thaliana’s MCTP4 structure using prediction tools and coarse grained simulations of transmembrane domain dynamics
Source: PLoS One. 2025 Jul 15;20(7):e0326993. doi: 10.1371/journal.pone.0326993 (PMC12262843; doi:10.1371/journal.pone.0326993)
Supplement: S1 Table — (PDF) [file pone.0326993.s001.pdf]

**Models**	**Tilt**	**Depth**
AF	15 ± 0°	27.2 ± 1.2 Å
AFM	40 ± 0°	31.2 ± 0.7 Å
OF	37 ± 4°	31.2 ± 0.8 Å
ESM	34 ± 0°	27.8 ± 2.4 Å
RF	72 ± 1°	17.8 ± 1.2 Å
TR	33 ± 2°	30.8 ± 1.1 Å
