# Supplementary figures and images for: Consensus structure prediction of A. thaliana’s MCTP4 structure using prediction tools and coarse grained simulations of transmembrane domain dynamics
Source: PLoS One. 2025 Jul 15;20(7):e0326993. doi: 10.1371/journal.pone.0326993 (PMC12262843; doi:10.1371/journal.pone.0326993)

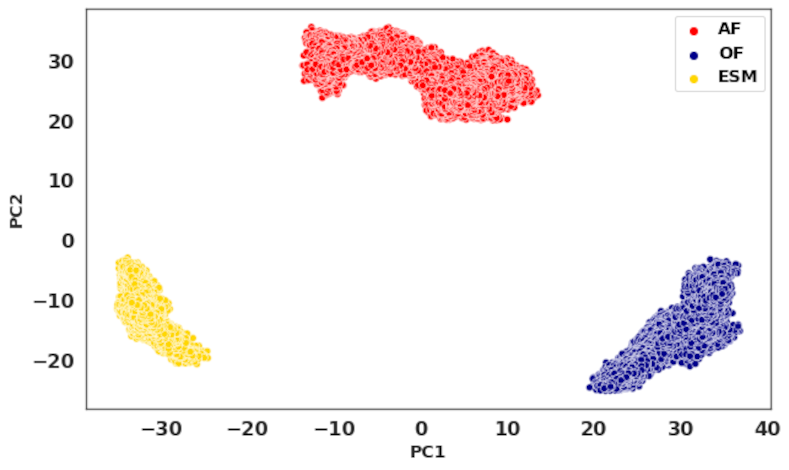

Supplement: S1 Fig — Each point represents an observation. Colors represent different models: Red for AF2, Navy Blue for OMEGA, and Gold for ESM. (TIF) [file pone.0326993.s002.tif]

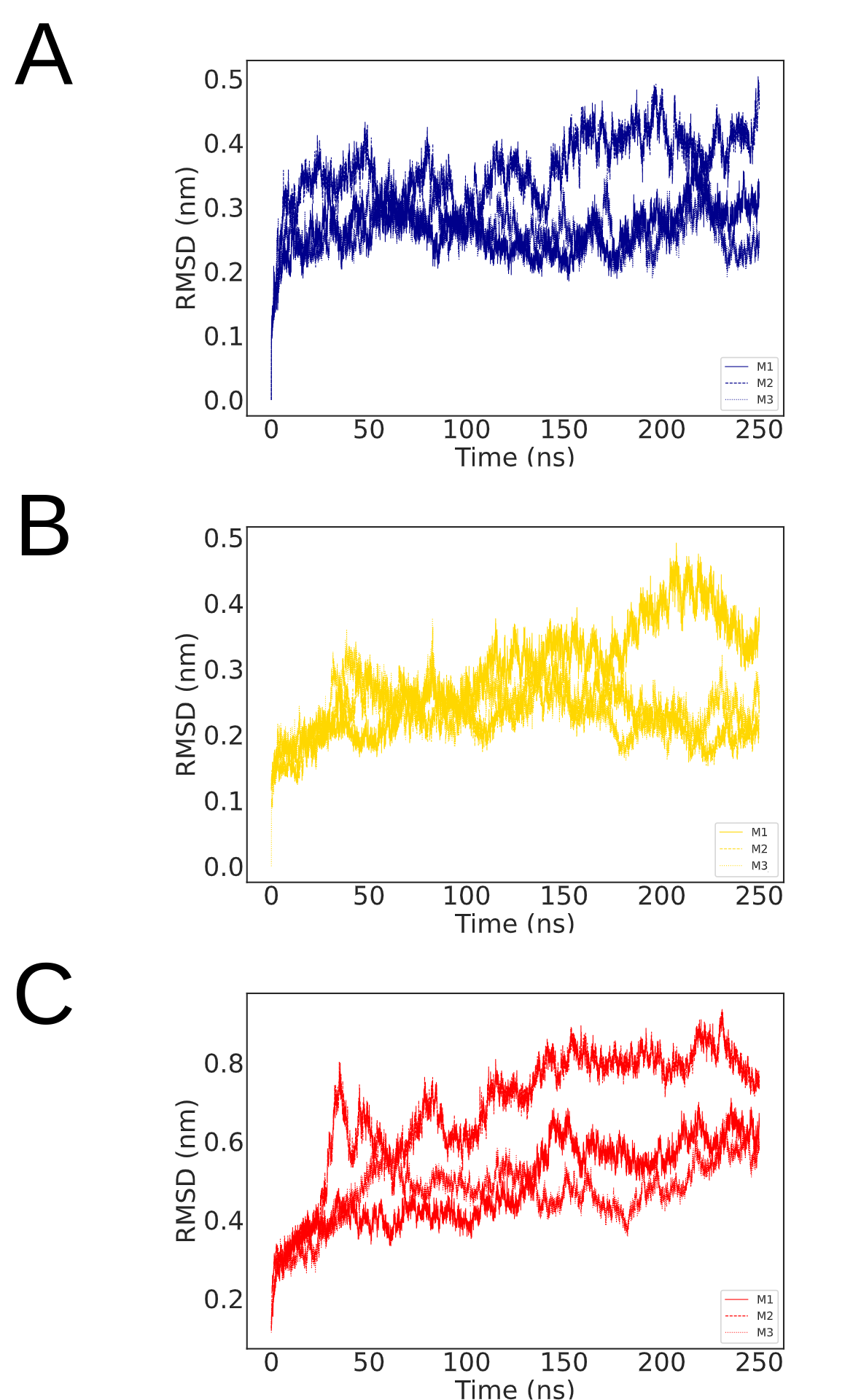

Supplement: S2 Fig — Red for AF2, Navy Blue for OMEGA, and Gold for ESM. (TIF) [file pone.0326993.s003.tif]

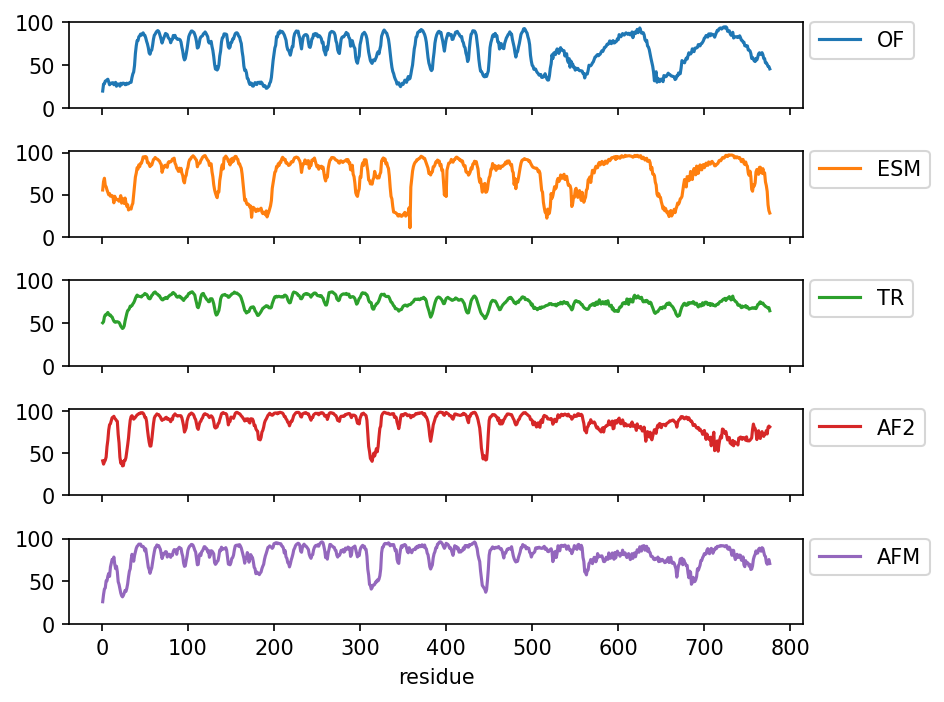

Supplement: S3 Fig — The curves of different colors represent models predicted by various prediction methods: AlphaFold (AF, red), OmegaFold (OF, blue), TR (green), ESM (orange), and AlphaFold Multimer (AFM, purple). (TIF) [file pone.0326993.s004.tif]

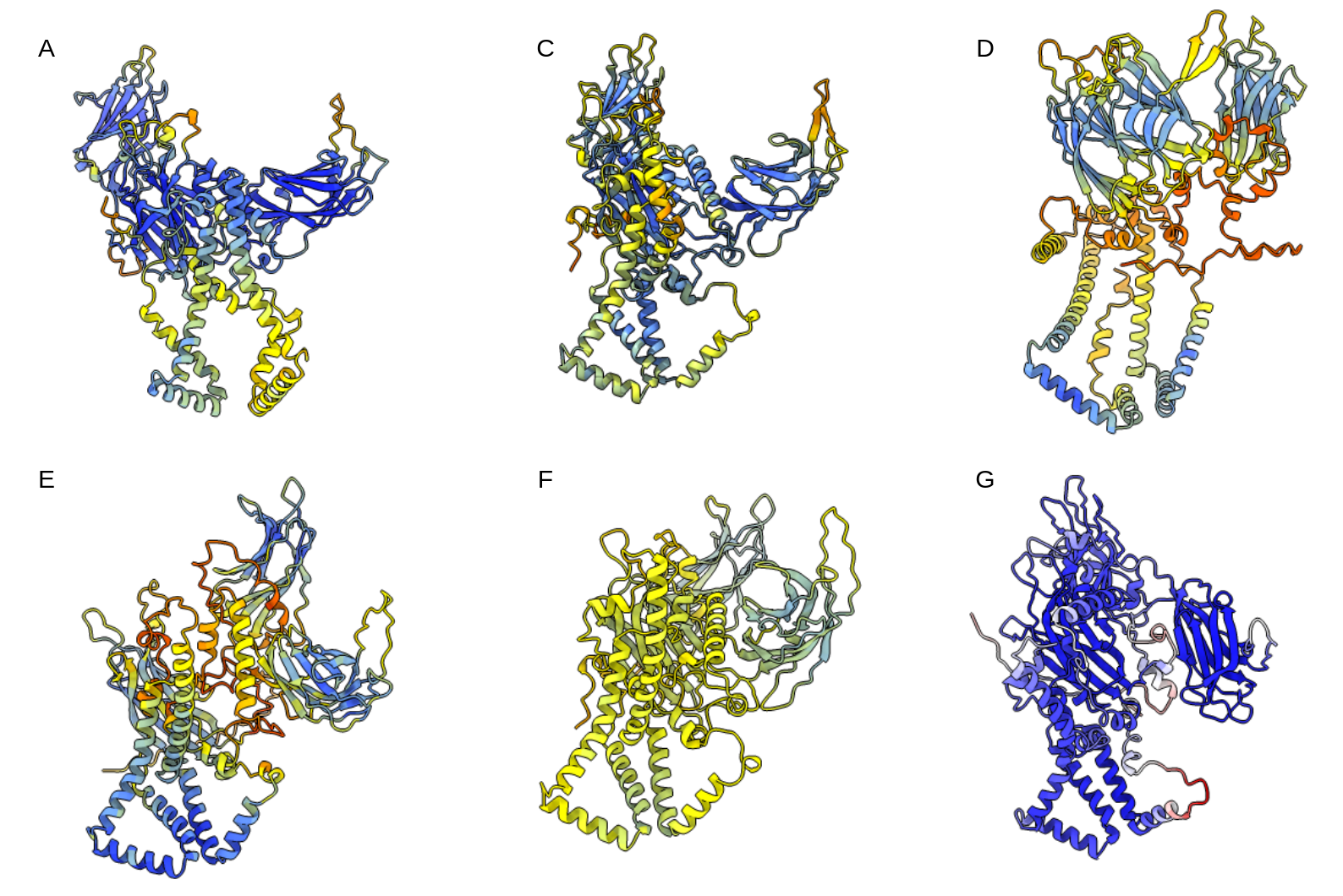

Supplement: S4 Fig — Panel G displays the prediction made by RosettaFold, colored on the error estimate in the Å (RMSD) metric. This metric categorizes structural predictions into three levels of confidence: blue for very confident, red for less confident, and white for areas lacking confidence. (TIF) [file pone.0326993.s005.tif]

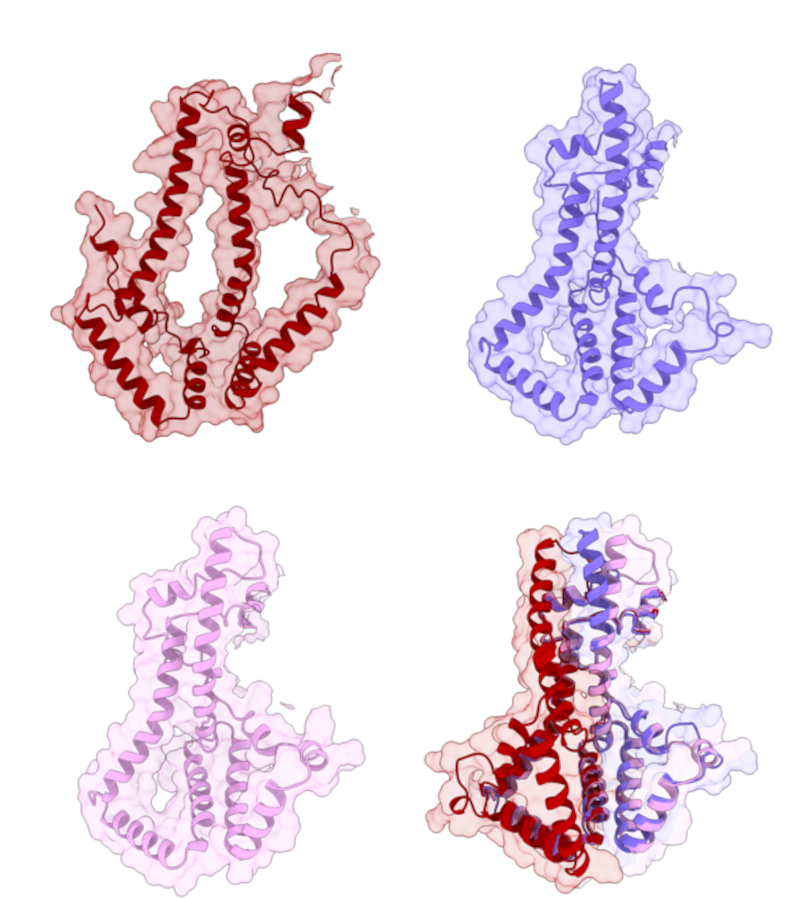

Supplement: S5 Fig — The AF3 model shows structural TMR similarities with the Omegafold model, while Boltz-1 and Chai-1 align with the consensus conformations observed in ESM, AFM, TR, and RF models. (TIF) [file pone.0326993.s006.tif]

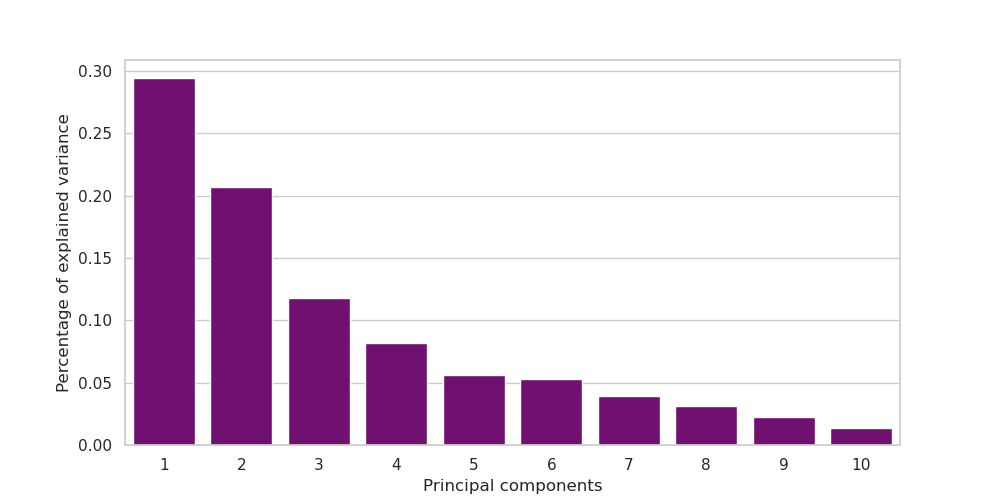

Supplement: S6 Fig — (TIF) [file pone.0326993.s007.tif]

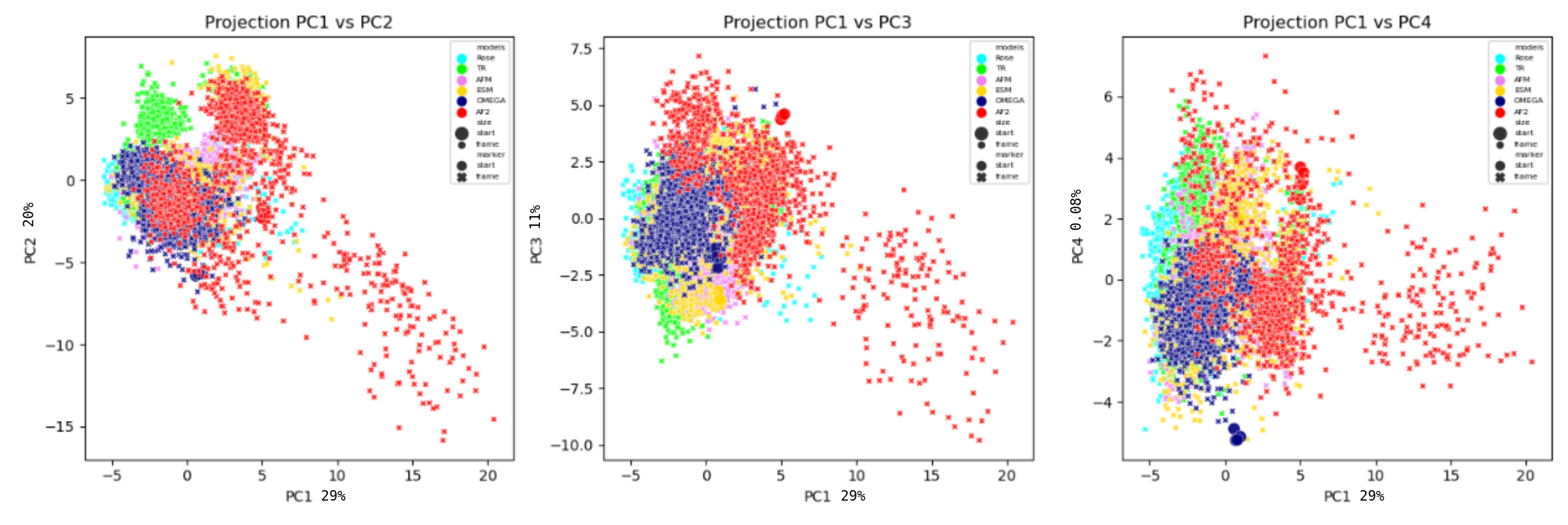

Supplement: S7 Fig — Each point represents an observation. Colors represent different models: Aqua for Rose, Lime Green for TR, Violet for AFM, Gold for ESM, Navy Blue for OMEGA, and Red for AF2. (TIF) [file pone.0326993.s008.tif]

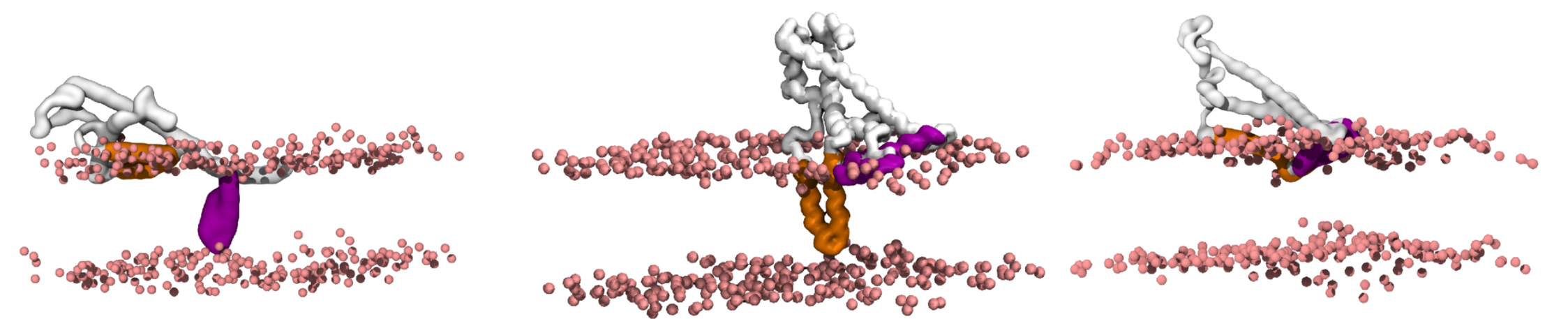

Supplement: S8 Fig — Center: HP1 domain emerging from the membrane. Right: Both HP1 and HP2 domains emerging from the membrane. These emergences from the membrane are observed in some replicas, regardless of the system in CG simulations. For our analysis, we chose not to consider these instances. Other predictive tools, such as PSIPRED [48] or DREAMM [49], indicate that this domain remains inside the membrane. When one or both domains emerge, they never re-enter the membrane during the simulation. (TIF) [file pone.0326993.s009.tif]

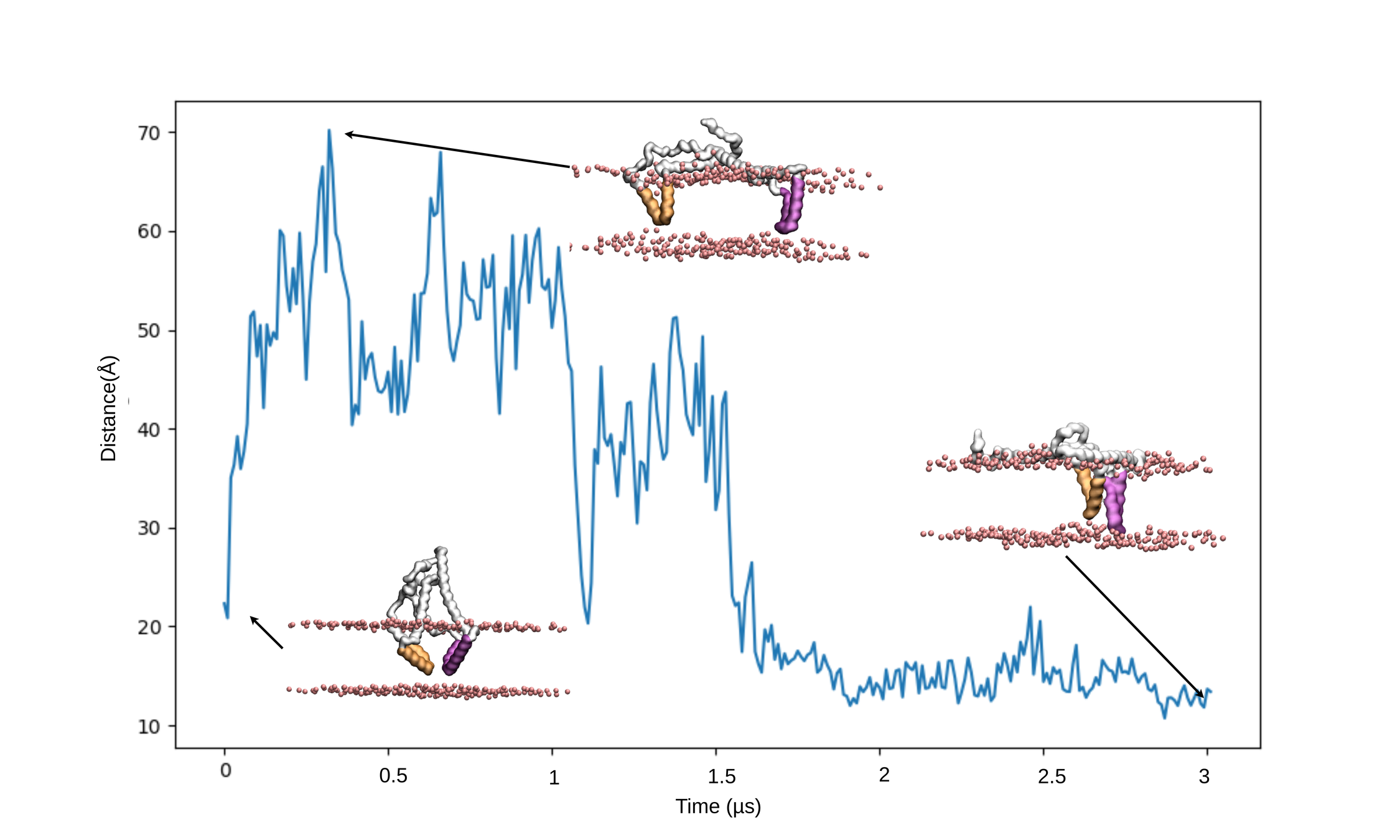

Supplement: S9 Fig — (TIF) [file pone.0326993.s010.tif]

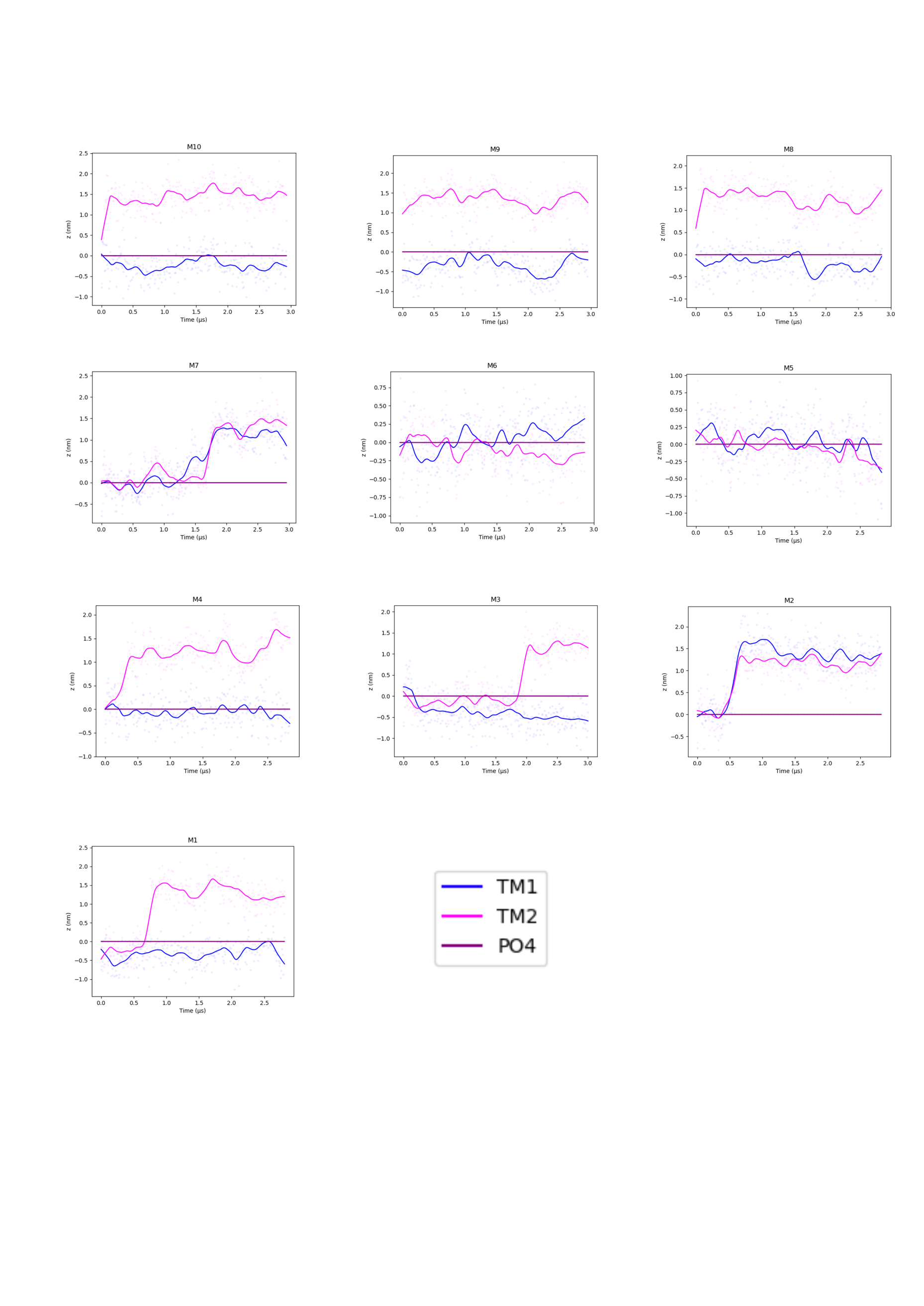

Supplement: S10 Fig — (TIF) [file pone.0326993.s011.tif]

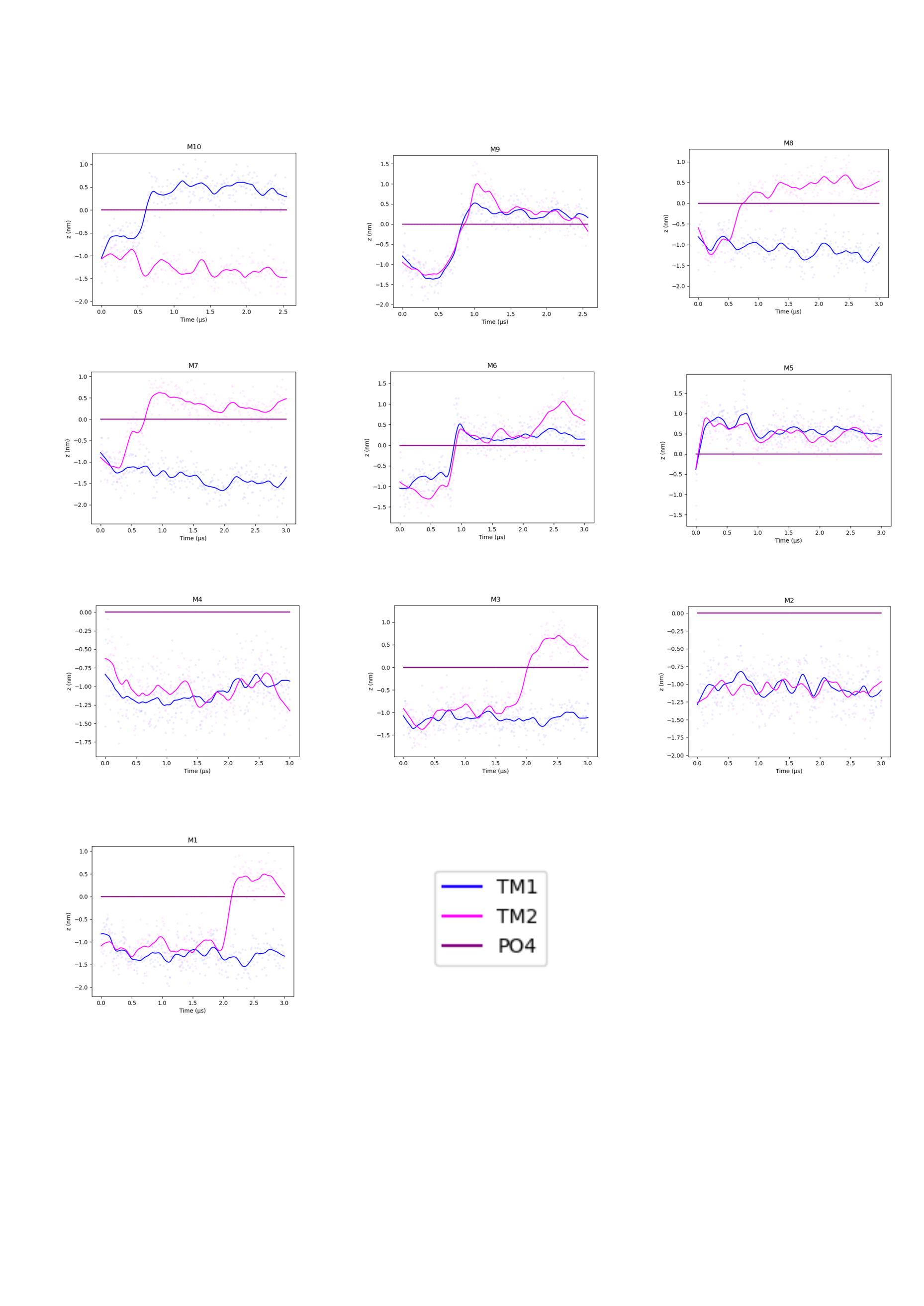

Supplement: S11 Fig — (TIF) [file pone.0326993.s012.tif]

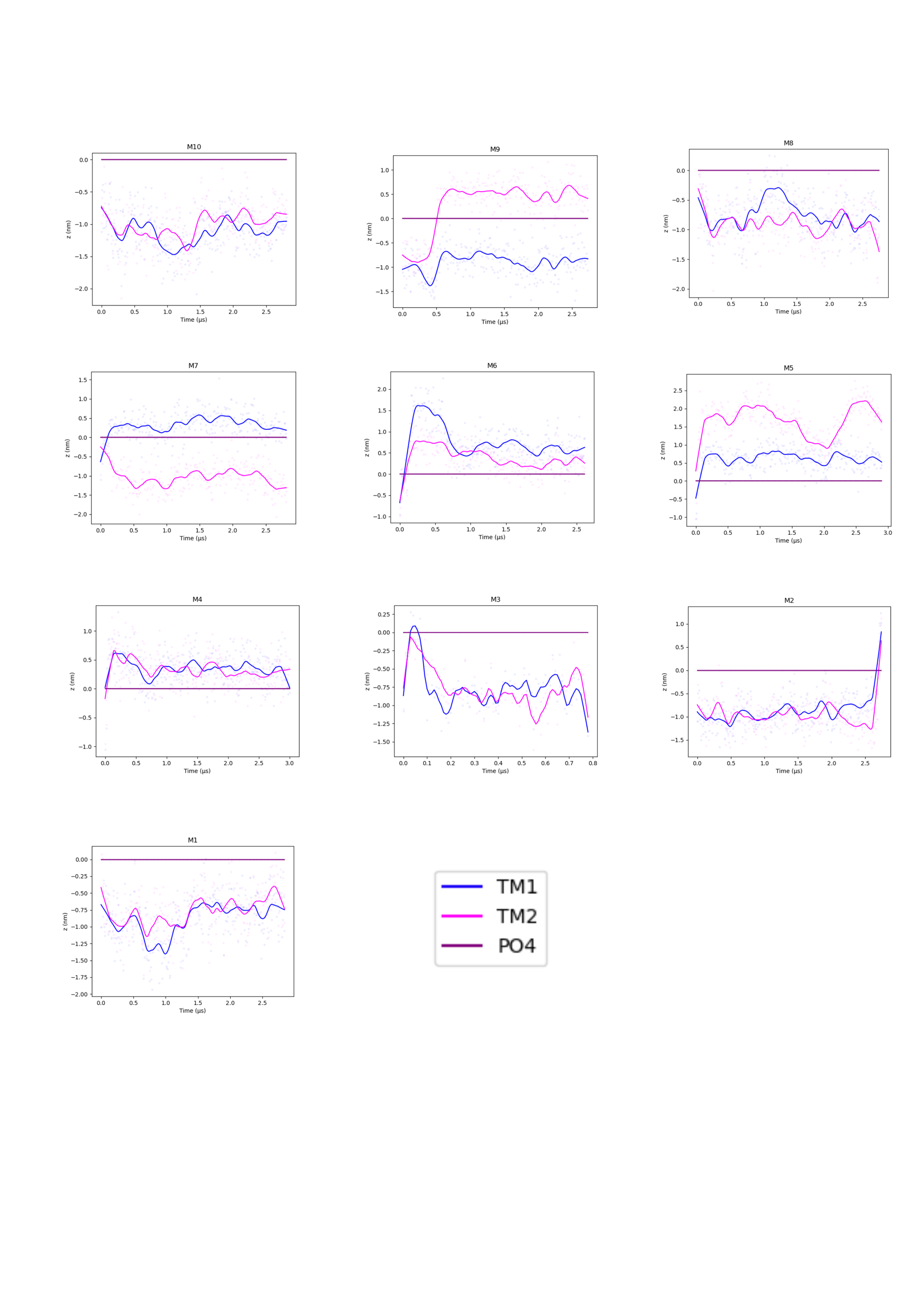

Supplement: S12 Fig — (TIF) [file pone.0326993.s013.tif]

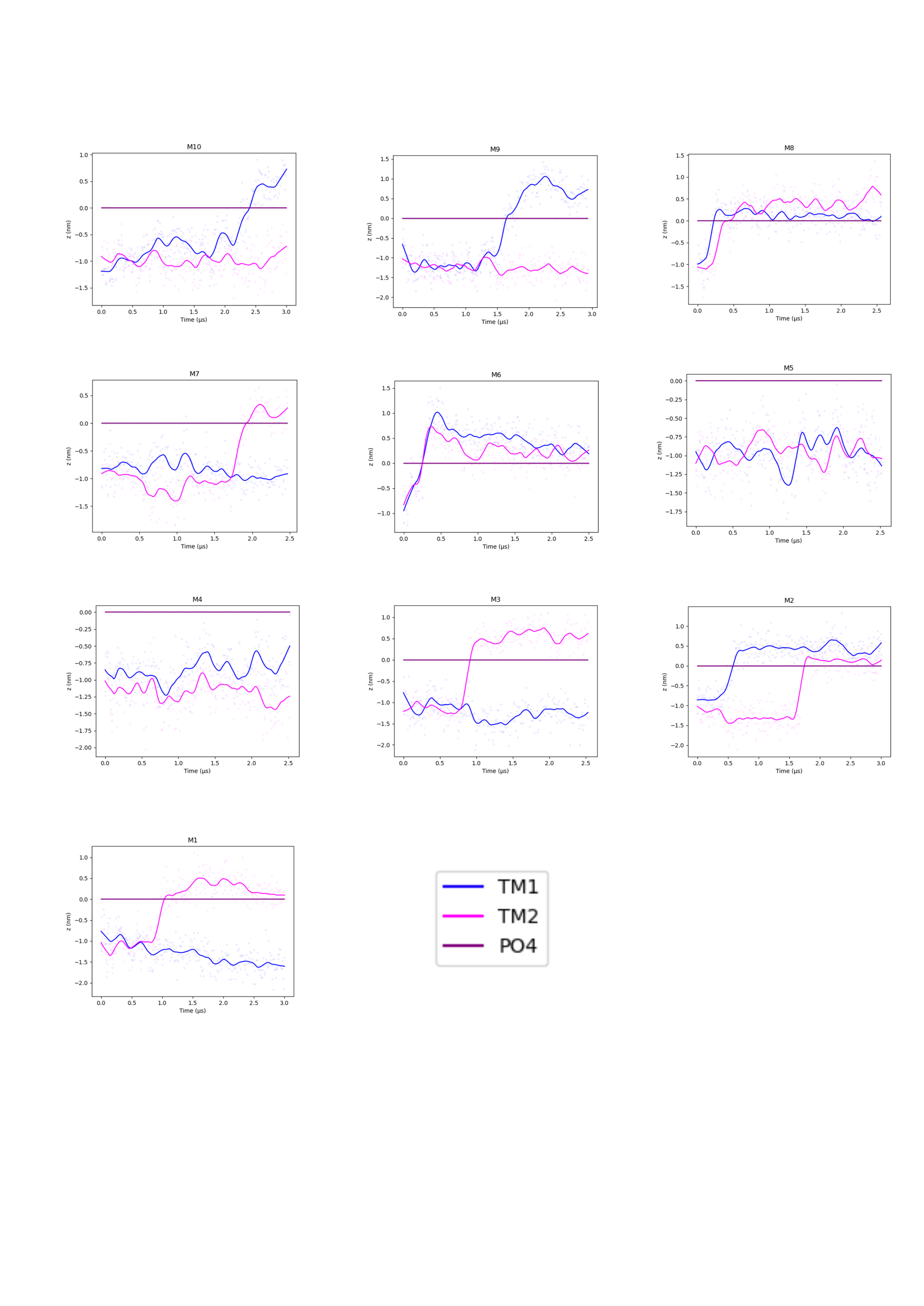

Supplement: S13 Fig — (TIF) [file pone.0326993.s014.tif]

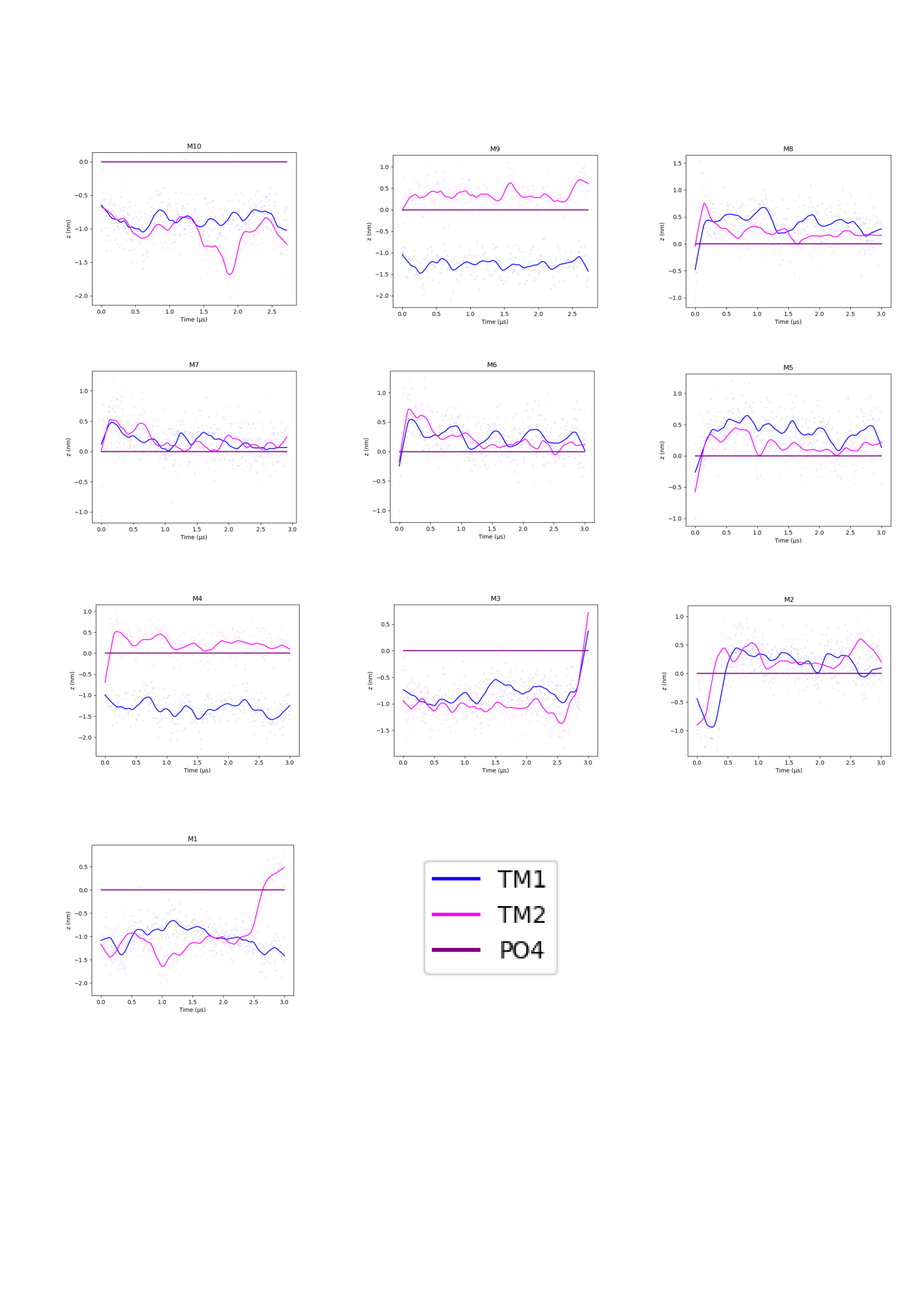

Supplement: S14 Fig — (TIF) [file pone.0326993.s015.tif]

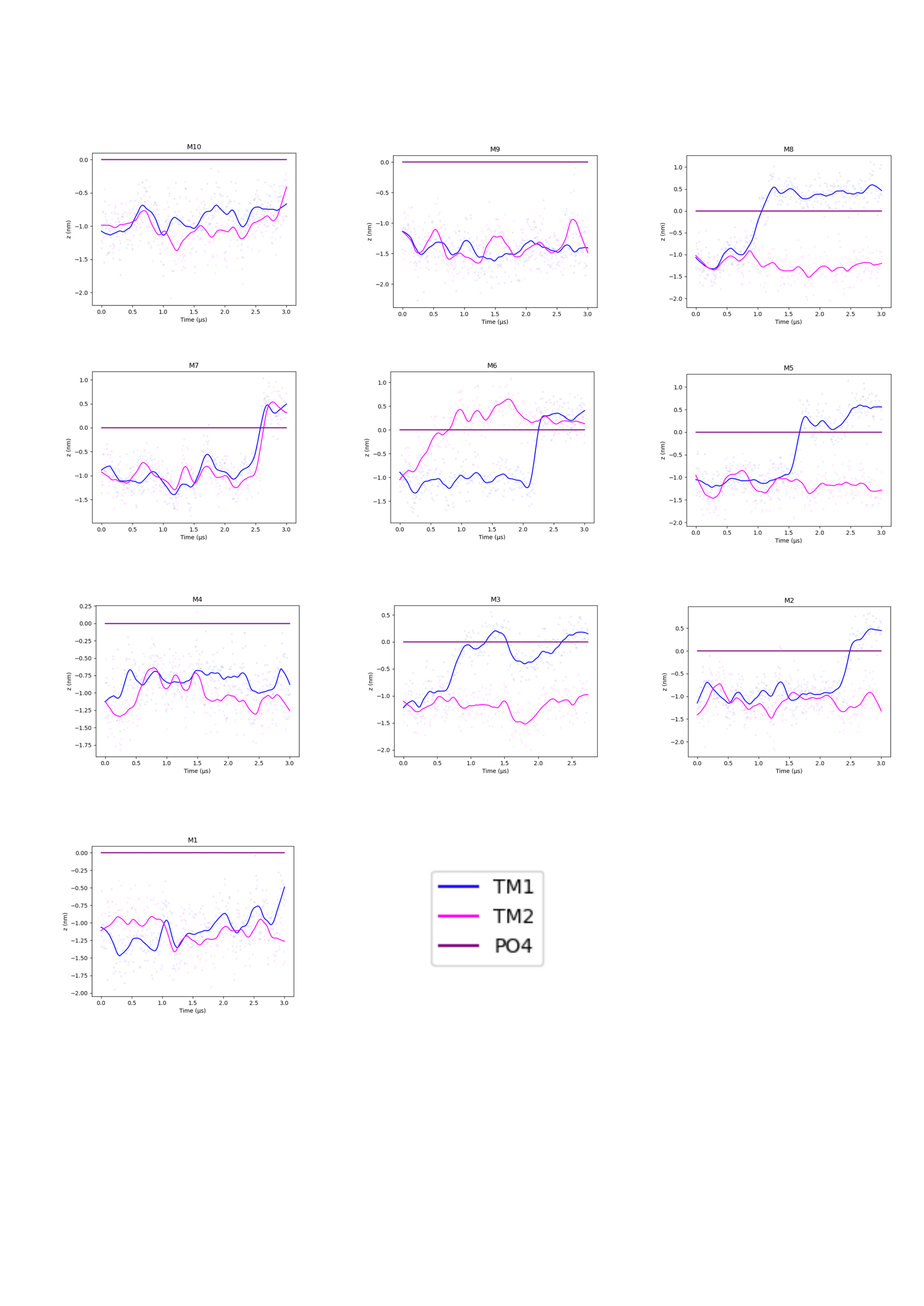

Supplement: S15 Fig — (TIF) [file pone.0326993.s016.tif]

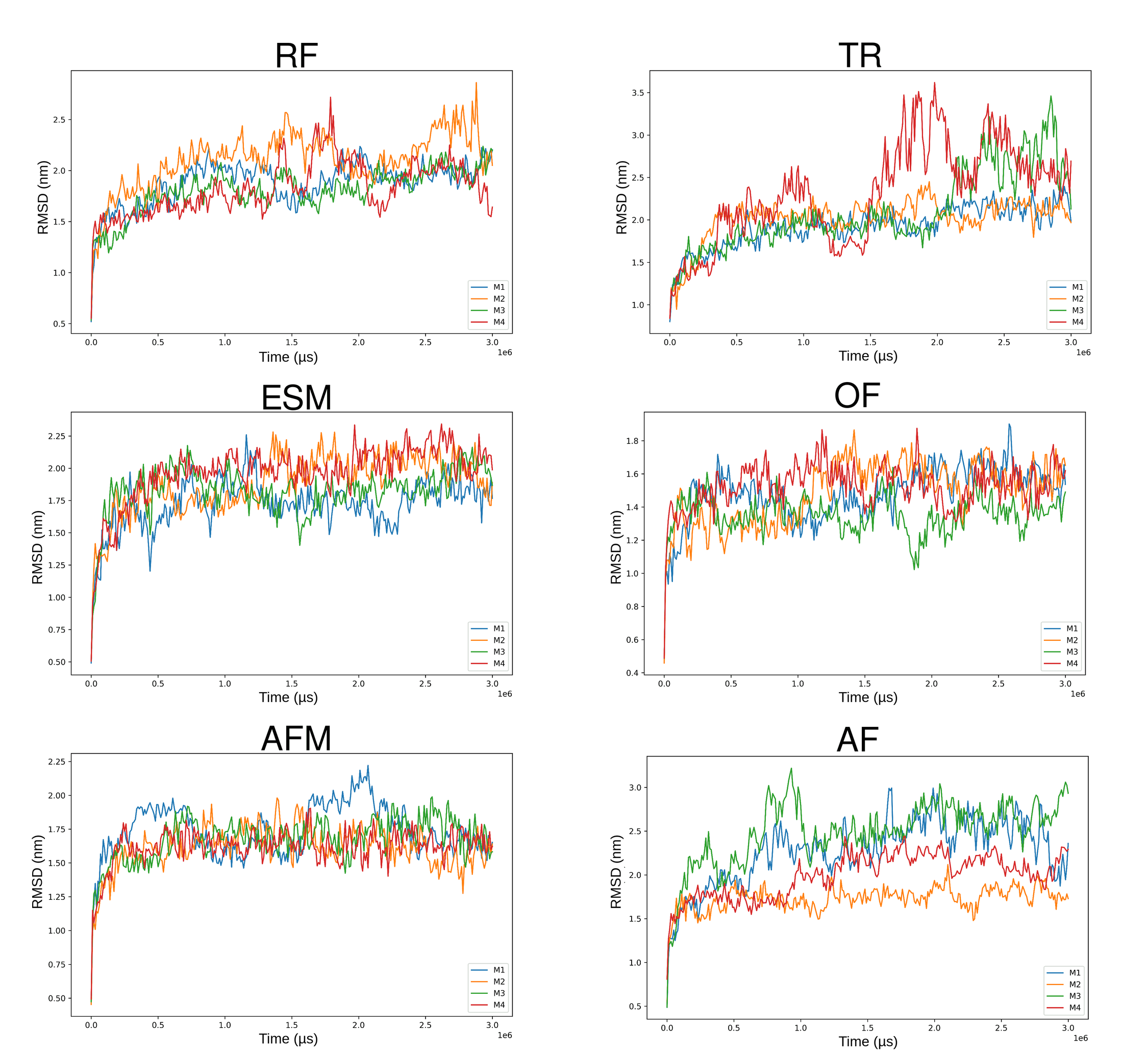

Supplement: S16 Fig — (TIF) [file pone.0326993.s017.tif]

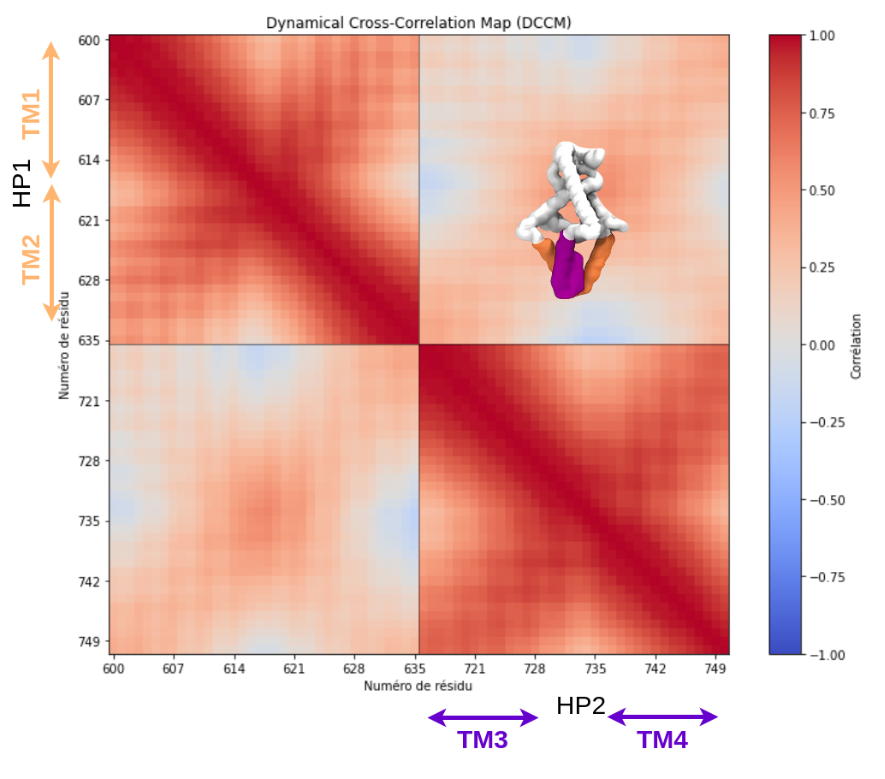

Supplement: S17 Fig — The analysis reveals that the two helices within each transmembrane hairpin (HP1 and HP2) move as coherent units, showing positive correlation within each hairpin. Between HP1 and HP2, a weaker positive correlation is observed, consistent with the formation of cluster D identified in the PCA analysis. (TIF) [file pone.0326993.s018.tif]

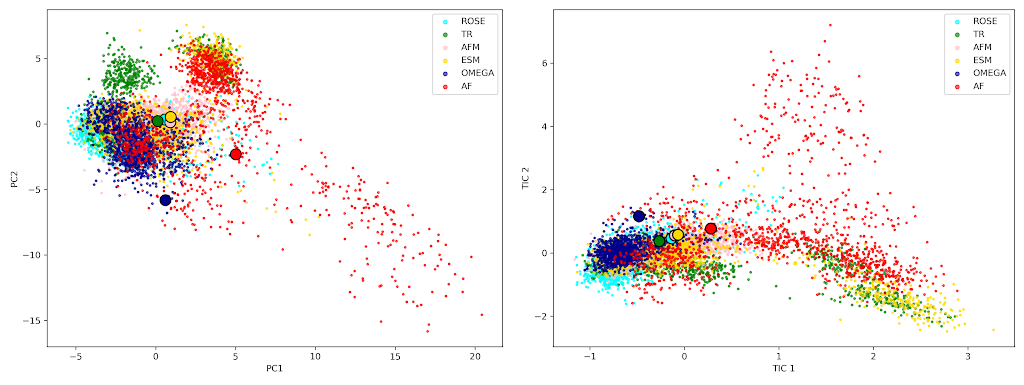

Supplement: S18 Fig — (TIF) [file pone.0326993.s019.tif]

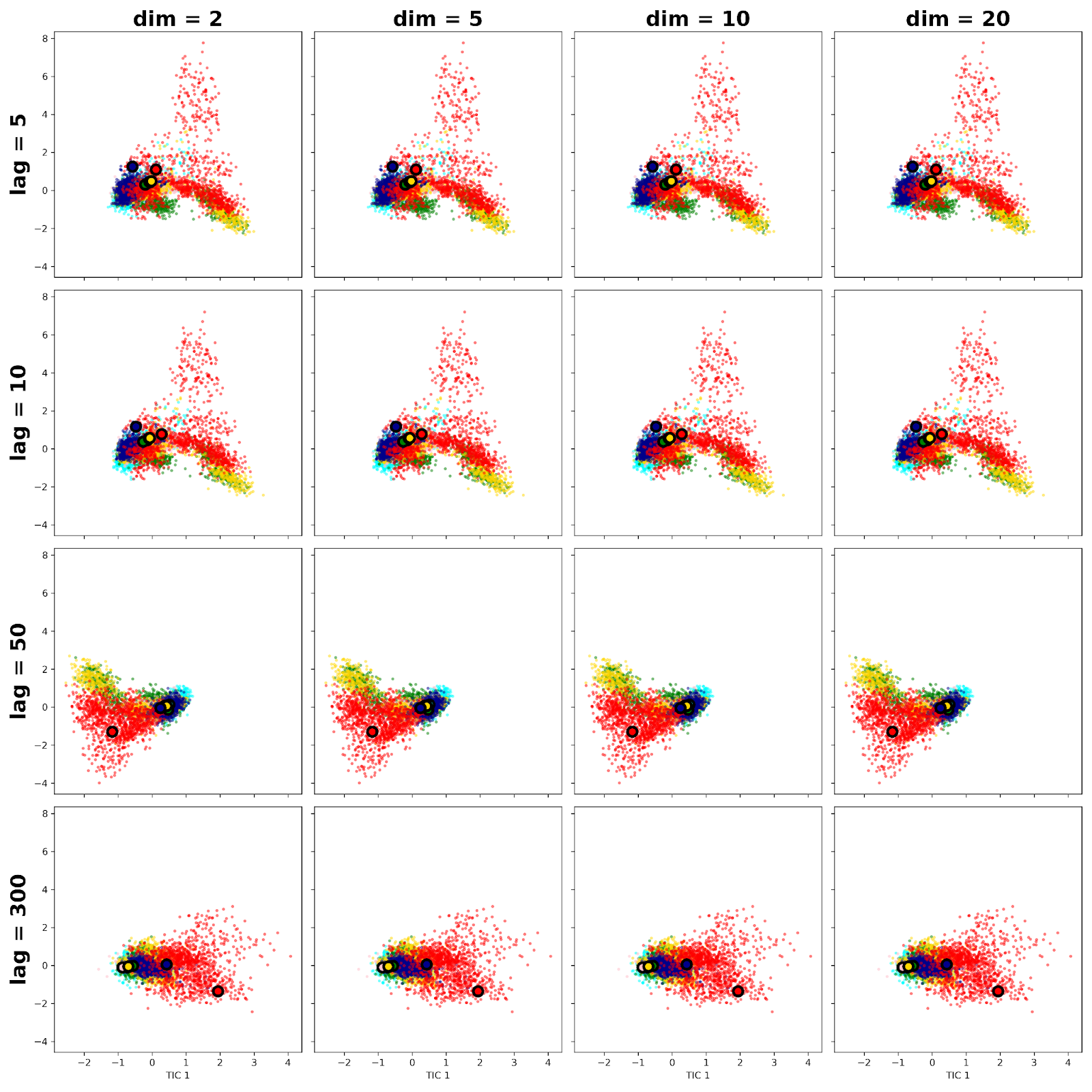

Supplement: S19 Fig — (TIF) [file pone.0326993.s020.tif]
