# Supplementary material for: Consensus structure prediction of A. thaliana’s MCTP4 structure using prediction tools and coarse grained simulations of transmembrane domain dynamics
Source: PLoS One. 2025 Jul 15;20(7):e0326993. doi: 10.1371/journal.pone.0326993 (PMC12262843; doi:10.1371/journal.pone.0326993)
Supplement: Attachment — Submitted filename: Sritharan et al.pdf [file pone.0326993.s021.pdf]

The manuscript by Sritharan et al. presents structural models of the Arabidopsis MCTP4 ER-anchor transmembrane region (TMR) domain using several deep learning-based structure prediction methods. Additionally, the authors performed molecular dynamics (MD) simulations to explore the behavior of the TMR within a lipid bilayer environment. The approach holds promise for capturing the structural complexity and dynamism of the MCTP4 TMR domain within an ER-mimicking bilayer. While the manuscript is well-constructed and the results are interesting, several points need to be addressed before the manuscript can be considered for publication.

Major Comments:

The authors employed the CHARMM36m force field for all-atom (AA) MD simulations. However, the reason behind choosing this particular force field should be clarified, especially in the context of lipid–protein interactions.

The authors are encouraged to perform a dynamical cross-correlation analysis to gain insights into the most significant collective motions of the TM helices within the lipid bilayer.

The AA MD simulations are currently limited to 250 ns, which may not be sufficient to fully capture the equilibrium behavior of the system. Extending the AA MD simulations to at least 1 μs would significantly strengthen the robustness of the conclusions.

The introduction and discussion would benefit from incorporating recent references on structure prediction and MD simulations of MCTP4 ER-anchor TMR domains, as well as a clearer articulation of how this study advances the current understanding of MCTP4 structure and function.

Minor Comments:

The figure numbers are missing in several places within the main text, making it difficult to follow the results.

The "Jupyter Notebook" section in the Materials and Methods should be removed, and the corresponding GitHub link can be provided under the Data Availability section instead.

Typos and grammatical issues should be carefully checked.
