# Supplementary material for: Consensus structure prediction of A. thaliana’s MCTP4 structure using prediction tools and coarse grained simulations of transmembrane domain dynamics
Source: PLoS One. 2025 Jul 15;20(7):e0326993. doi: 10.1371/journal.pone.0326993 (PMC12262843; doi:10.1371/journal.pone.0326993)
Supplement: Attachment — Submitted filename: Lettre reviewer.pdf [file pone.0326993.s022.pdf]

We sincerely thank the reviewers for their thorough review of our manuscript and for their constructive and insightful comments, which have greatly contributed to improving its quality. We have carefully considered all remarks and revised the manuscript accordingly to address the points raised. In this revised version, we provide additional explanations, strengthen our rationale, and, where relevant, include new analyses (e.g. dynamic cross-correlation) to better substantiate our conclusions. We believe these changes significantly enhance the manuscript and hope it now fully meets the reviewers' expectations.

Reviewer #1: The manuscript titled "Prediction of A. thaliana's MCTP4 Structure using Deep Learning-Based Tools and Exploration of Transmembrane Domain Dynamics using Coarse-Grained Molecular Dynamics Simulations" investigates the structural dynamics of the Arabidopsis thaliana MCTP4 protein. The study employs structure prediction methods and coarse-grained molecular dynamics simulations to analyze the transmembrane region (TMR) of MCTP4. This study advances the understanding of plant membrane protein structures and demonstrates the necessity of integrating multiple computational approaches for accurate structural modeling.

This paper requires major revisions before it can be considered for publication. The issues raised in the questions highlight key concerns regarding the interpretation of structural convergence, the handling of molecular dynamics results, and the reliability of deep learning-based predictions in the context of membrane proteins**.** The authors should address these points thoroughly and provide stronger justifications for their methodological choices. I recommend resubmitting the manuscript after a careful revision that clarifies these aspects.

Introduction

1. While deep learning plays a role in the structure prediction methods used, explicitly stating 'Deep Learning-Based Tools' in the title may be unnecessary. Since AlphaFold, RosettaFold, and similar tools are well-established in the field, I suggest simplifying the title to focus on the structural prediction and molecular dynamics aspects without emphasizing deep learning explicitly.

We acknowledge the reviewer's perspective on the title. While AlphaFold and RosettaFold are well-established, we utilized a range of deep learning-based tools, each providing different conformations (as detailed in the supplementary materials).
We propose to change the title to reduce the emphasis on deep learning: "Consensus structure prediction of A. thaliana's MCTP4 structure using prediction tools and coarse grained simulations of transmembrane domain dynamics".

2.The authors mention that deep learning models such as Alphafold, RosettaFold, ESMFold, and OmegaFold have limitations due to the underrepresentation of membrane proteins in training datasets. However, the study relies on these tools to model the transmembrane region (TMR) of MCTP4. Given that deep learning models are inherently biased toward their training data, how do the authors quantitatively ensure that the models are not influenced by this bias? Have the authors benchmarked the results against experimentally solved membrane proteins of similar topology to assess reliability?

We thank the reviewer for this important point.
We fully agree that deep learning models are inherently influenced by the data they are trained on, and that membrane proteins remain underrepresented in many structural databases. However, our study addresses this limitation in several ways:
First, since no experimentally resolved structure is available for MCTP4 or closely related homologs [1], we adopted a consensus-based modeling strategy. We used multiple state-of-the-art prediction tools (AlphaFold, AlphaFold Multimer, RoseTTAFold, Tr-rosetta,ESMFold, and OmegaFold), each relying on different architectures and data sources. For instance, AlphaFold and RoseTTAFold depend heavily on multiple sequence alignments (MSA), while ESMFold and OmegaFold do not require MSAs and are trained on vast protein sequence datasets. This diversity in model design helps reduce shared biases.

Moreover, by systematically comparing the structural outputs from each tool, we were able to identify consistent features particularly in the transmembrane regions. These convergences across models increased our confidence in the predicted topology, even in the absence of direct experimental data.

In addition, to further test the stability and plausibility of these predicted structures, we performed molecular dynamics (MD) simulations. These simulations rely on physics-based principles rather than training data, and thus offer an independent means of validating the structural integrity of the transmembrane regions.

Finally, while benchmarking against experimentally solved membrane proteins of similar topology would indeed provide an extra layer of validation, such an analysis falls beyond the scope of this study, which focuses on a protein family lacking any resolved structures. We have clarified this in the revised manuscript to ensure the reader does not mistakenly perceive our modeling approach as uncritical or biased.

Original text:

“One such limitation is that membrane proteins are underrepresented in the training datasets, which could lead to uncertain models. On the other hand, new methods based on language models, such as the one developed by Lin et al. (2022), are not limited by experimental data."

Revised text :

Unlike AlphaFold, which relies heavily on multiple sequence alignments (MSA) and experimentally determined structures from the Protein Data Bank (PDB), ESMFold and Omegafold leverages a large-scale language model trained on vast and diverse sequence datasets, including metagenomic sequences for ESMFold. This approach makes both models less dependent on the availability of experimentally solved structures, such as those of membrane proteins.

[1] Membrane Tethering in Plant Intercellular Communication : Structure-Function of Multiple C2 domains and Transmembrane Region Proteins (MCTP) at Plasmodesmata ER-PM Membrane Contact Site; Thesis, Jules Petit, https://theses.hal.science/tel-03789611.

3.The study combines coarse-grained (Martini 3) and all-atom (CHARMM36) molecular dynamics simulations to validate the conformational dynamics of the predicted transmembrane region. However, coarse-grained models inherently reduce atomic detail, while all-atom simulations are computationally expensive and limited in timescales. How do the authors ensure that coarse-grained simplifications do not overlook key molecular interactions essential for membrane stability? Conversely, are the all-atom simulations long enough to capture biologically relevant conformational dynamics?

In our approach we use the CG model to explore the conformation of MCTP4' TMR domain. CG models, such as Martini 3, sacrifice atomic detail to enable extensive conformational sampling over longer timescales, which is a key strength for capturing large-scale dynamics. In our study, CG simulations revealed significant mobility of the transmembrane hairpin within the membrane, a phenomenon that would be difficult to observe with AA simulations alone due to their time scale limitations.
We fully agree with the reviewer's assessment that all-atom simulations carry a prohibitive computational cost, making them impractical as the primary method for exploring transmembrane protein conformational dynamics. This limitation precisely motivated our two-steps strategy, which we would like to clarify.

To assess the quality of the resulting models we then turn to all-atom (AA)

simulations. We conducted nine independent AA runs with CHARMM36: using three starting structures (ESMFold, AlphaFold, OmegaFold) and three replicas each with randomized velocities. This multi-structure, multi-replica approach confirmed the stability of the transmembrane hairpin in the membrane, testing if it exists as seen in some CG simulations. Despite their shorter timescales, these AA simulations validated the hairpin's structural integrity. We propose to modify the text to clarify this point.

In original text :

"Finally, we employed molecular dynamics (MD) simulations with the Martini 3 coarse-grained and Charmm36 all-atom force fields, along with principal component analysis."

Revised text :

Finally, we employed a two-tiered molecular dynamics (MD) simulation strategy: (1) Martini 3 coarse-grained simulations for extensive conformational sampling of the transmembrane domain, and (2) nine independent Charmm36 all-atom simulations (three starting structures with three replicas each) to verify structural stability and evaluate potential helix extrusion events. This complementary approach was coupled with principal component analysis to characterize the conformational landscape.

4.The introduction states that MCTP proteins act as ER-PM tethers and are localized at plasmodesmata, potentially regulating intercellular transport. However, the focus of this study is on predicting the structure of the MCTP4 transmembrane region. Given that the tethering function likely depends on interactions between the transmembrane and cytoplasmic domains, how does studying the isolated TMR provide meaningful insights into its function? Would not modeling the entire MCTP4 protein, including C2 domains, provide a more biologically relevant structural interpretation?

We thank the reviewer for this valuable suggestion and agree there is a lack of justification on why we want to study the transmembrane part of the reticulum. Accordingly, we have incorporated references to a recent work of Patrick Li et al., which highlights the critical role of MCTP4 in stabilizing endoplasmic reticulum (ER) connections across plasmodesmata [1]. This study shows that mutations in the transmembrane (TMR) domain destabilize these channels, leading to their complete closure, underscoring the functional importance of the TMR. We first get 3 different conformations of TMR which are not elucidated by experiment by using deep learning and then use physics-based methods to explore conformation by using Martini 3 simulation. By characterizing the collective motions and stability of the TM helices within the lipid bilayer, our study elucidates the dynamic of the TM, offering a more

comprehensive view of MCTP4's role in ER-plasmodesmata connectivity. This advances the current understanding by linking structural predictions and dynamic simulations to the functional observations reported by Li et al., thus providing a mechanistic basis for MCTP4's stabilizing effect.
We also included a reference to a recent study where Martini coarse-grained molecular dynamics (MD) simulations were used to demonstrate that the C2 domains of MCTP4 interact with PI4P lipids **[2]**, providing insights into their membrane-binding behavior.

Original text:

While C2 domains are relatively conserved and the 3D structure is more easily accessible, the TMR presents a complex domain organization that has not yet been elucidated.

Revised text :
The cytosolic C2 domains were recently shown, through both Martini simulations and experimental evidence, to interact with PI4P lipids. These interactions support a role for MCTP4 in regulating intracellular trafficking via its C2 domains. In parallel, the transmembrane region (TMR) has been identified as critical for stabilizing ER connections at plasmodesmata, with mutations in this region disrupting ER-PM continuity and leading to pore closure [2]. Despite this functional importance, the TMR remains structurally unresolved, and its three-dimensional organization is poorly characterized.

[1] http://dx.doi.org/10.2139/ssrn.4682263
[2] DOI: 10.1126/science.adn4630

Materials and Methods

1.The authors state that they compared results from various deep learning-based prediction tools (Alphafold, RosettaFold, ESMFold, OmegaFold, etc.) to ensure reliability. However, the choice of models seems to rely primarily on convergence across tools, rather than on an objective experimental validation metric. Given that membrane proteins are underrepresented in training datasets for these models, how do the authors ensure that the observed convergence is not merely a reflection of shared biases across deep learning models rather than genuine structural accuracy? Would not benchmarking against experimentally solved membrane protein structures be a stronger validation approach?

We thank the reviewer for raising this important point. Our study adopts a prospective approach to investigate the structural dynamics of the MCTP4 transmembrane domain, a protein for which no experimentally solved structure is currently available [3]. Given this context, our reliance on

convergence across multiple deep learning-based prediction tools (AlphaFold, RosettaFold, ESMFold, OmegaFold, TR-Rosetta) is intended to maximize the robustness of our predictions in the absence of experimental data, rather than to assume absolute structural accuracy. While we acknowledge the potential for shared biases among these models, we use a physics-based method, molecular dynamics (MD) simulations, to mitigate this risk. To address the reviewer's concern and clarify our approach, we propose revising the text in the Materials and Methods section as follows:

Original text: 'We compared results from various deep learning-based prediction tools (AlphaFold, RosettaFold, ESMFold, OmegaFold, TR-rosetta) to ensure reliability.'

Revised text: 'In the absence of an experimentally determined structure for MCTP4, we employed a prospective approach by comparing predictions from multiple deep learning-based tools (AlphaFold, RosettaFold, ESMFold, OmegaFold, Tr-Rosetta.) to explore its conformational landscape. Convergence across these models, combined with MD simulations, was used to mitigate this risk.

[3] https://doi.org/10.1021/acs.jcim.3c01361

2.The authors describe the construction of transmembrane systems in a PIPC/PIPE bilayer membrane for CG-MD and a PLPE/PLPC lipid environment for AA-MD simulations. However, the ER-PM contact sites in plasmodesmata may contain additional lipid diversity, such as sterols or specific phosphoinositides. How do the authors justify these lipid choices, and have they considered whether these specific membrane environments sufficiently mimic the in vivo conditions of MCTP4 anchoring? Would varying the lipid composition affect the stability and interactions of the TMR?

The transmembrane region of MCTP interacts within the ER membrane. The precise lipid composition at plasmodesmata remains unknown, but the ER membrane composition in plants is well-characterized as primarily PIPC/PIPE [4]. Given this, we based our simulations on the ER lipid environment as a reasonable approximation. In Charmm36 the equivalent is PLPC/PLPE.

Original text
To investigate the stability of the TM helix, we performed simulations in PIPC/PIPE (80:20) bilayer membranes.

Revised text: 'The precise lipid composition of the ER at plasmodesmata remains unknown, but the ER membrane in plants is well-characterized as primarily PLPC/PLPE [4]. To investigate the stability of the TMR helix, we

therefore performed simulations in PLPC/PLPE (80:20) the bilayer membranes for AA-MD and PIPC/PIPE environments for CG-MD [5], as a reasonable approximation of the ER lipid environment anchoring MCTP4.'

[4]. Moreau, P. *et al.* Lipid trafficking in plant cells. *Prog. Lipid Res.* 37, 371–391 (1998).

[5] https://pubs.acs.org/doi/10.1021/acs.jctc.5b00209

3.The study employs coarse-grained Martini 3 simulations for long-timescale behavior and all-atom CHARMM36m simulations for finer structural insights. However, Martini 3 force fields have been known to sometimes overestimate protein stability in membranes due to their implicit treatment of hydrogen bonding and electrostatics. On the other hand, the 3-microsecond all-atom simulations may not fully capture slow conformational changes. Given these known issues, how do the authors ensure that Martini 3 does not artificially stabilize the TMR, and how do they justify that their AA-MD simulations are sufficiently long to explore meaningful structural transitions?

We acknowledge the potential limitations of Martini 3. It's still a recent improvement of the Martini 2. However, our coarse-grained simulations revealed variability in TMR behavior, in some instances, the transmembrane hairpin exhibited instability and exited the membrane early in the simulation, while in others, it remained embedded throughout. This heterogeneity suggests that Martini 3 does not overstabilize the TMR, as a systematic artifact would likely result in consistent stability across all replicas. To further distinguish between genuine protein dynamics and potential force field biases, we complemented the CG simulations with AA-MD simulations using the CHARMM36m force field, to distinguish between true protein behavior and Martini-induced artifacts. We explain and justify the all atom simulation in the previous question.

4.The authors used PCA to analyze transmembrane motions and subsequently K-Medoids clustering to classify different states. However, PCA reduces the dataset to a linear representation, which may not fully capture the nonlinear nature of membrane protein dynamics. How do the authors justify using PCA for highly heterogeneous conformational sampling? Given that alternative methods such as time-independent component analysis (tICA) or Variational Autoencoders (VAEs) have been more effective in capturing slow conformational dynamics, why were such approaches not considered?

We thank the reviewer for this insightful comment regarding the use of Principal Component Analysis (PCA) and alternative dimensionality reduction techniques. We initially selected PCA because it is a well-established and

widely used method to capture large-scale collective motions in molecular dynamics simulations [5]. Our goal was to identify dominant modes of motion across all models, particularly in the transmembrane regions. PCA remains a robust exploratory tool to reduce dimensionality and highlight the most significant variance in the data, especially when analyzing heterogeneous ensembles.

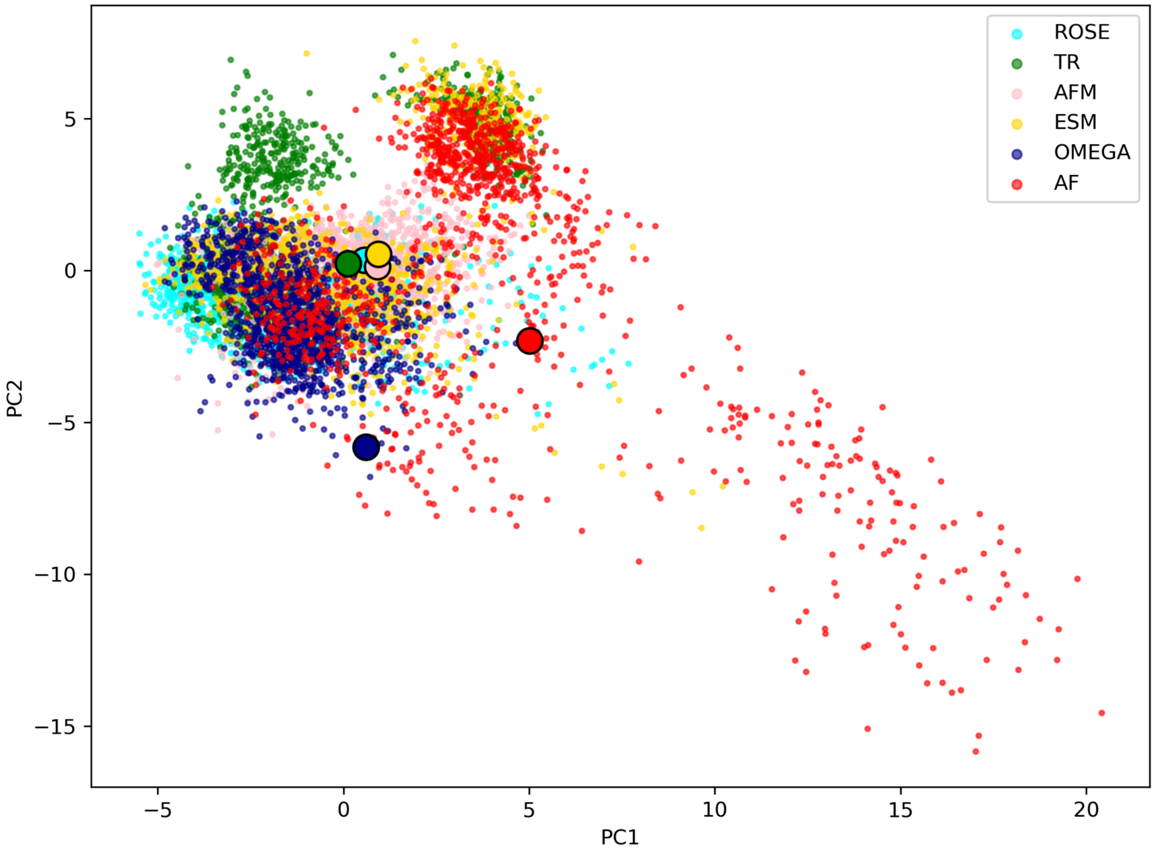

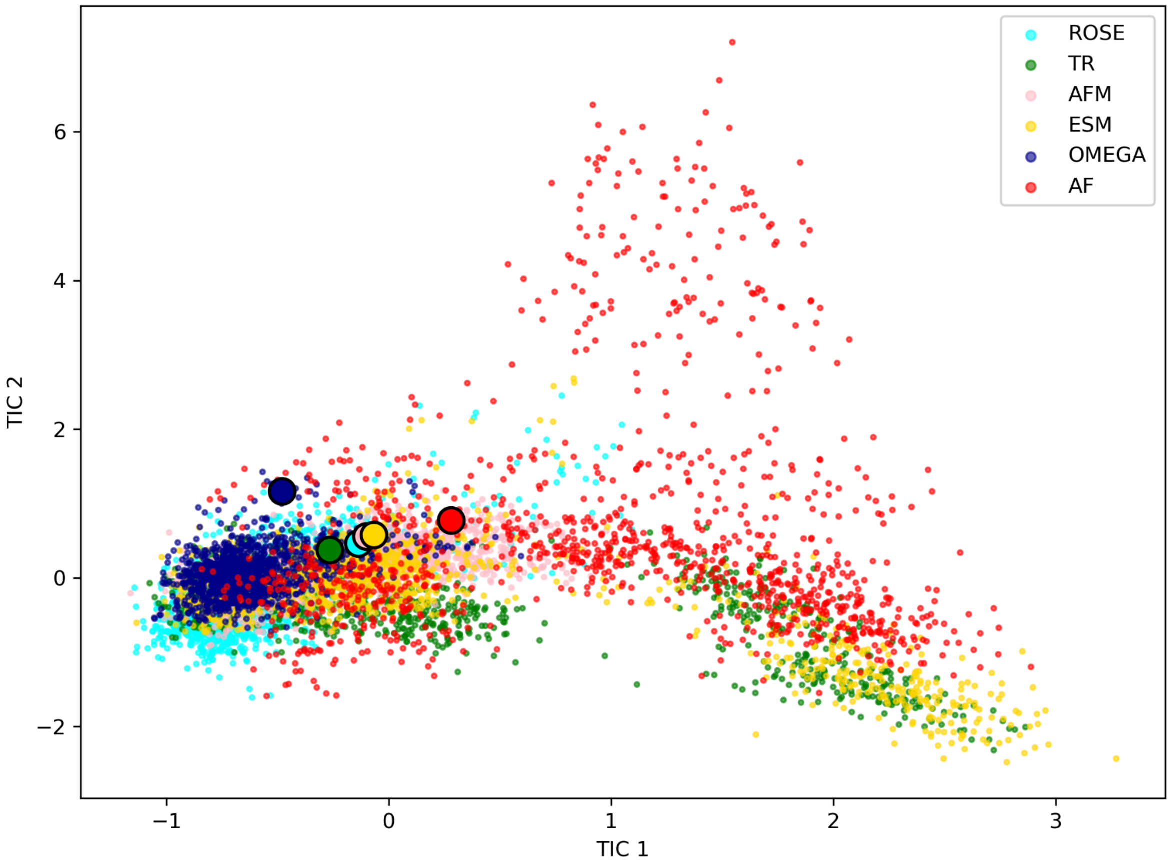

Figure : PCA tICA(lagtime 10)

To address the reviewer's concern about nonlinear dynamics, we also performed time-independent component analysis (tICA) on the concatenated trajectory (see additional figure above).

tICA showed a similar result to what was observed with PCA. one cluster which contains the majority of the conformation generated by the different trajectories and a second one which contains a part of trajectories initiated on 3 models (AF, ESM, TR). In the case of the AlphaFold (AF) trajectories, three main directions of motion could be identified. Overall, tICA provided results consistent with PCA, without uncovering additional slow modes.
We propose to put this result in supplementary material.

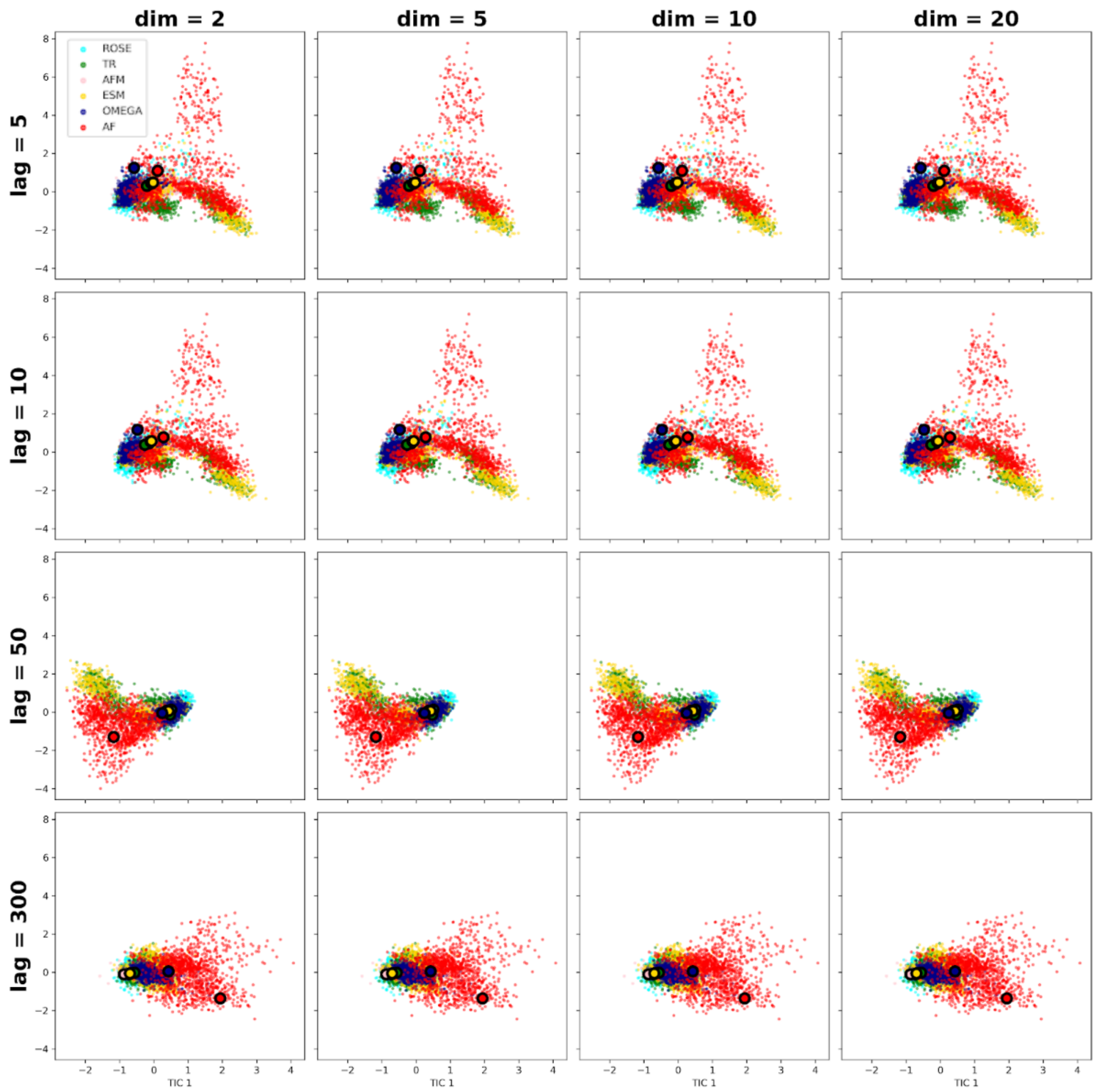

Figure : Projections of the first two tICA components for various lagtimes (5, 10, 50, 300) and dimensions (2, 5, 10, 20). Optimal metastable-state separation occurs at lagtime = 10 and dim = 2–10; beyond this, separation diminishes and higher dimensions offer no further improvement.

[5] Palma, J., & Pierdominici-Sottile, G. (2023). On the uses of PCA to characterise molecular dynamics simulations of biological macromolecules: basics and tips for an effective use. ChemPhysChem, 24(2), e202200491.

[6] Hao Wu, Feliks Nüske, Fabian Paul, Stefan Klus, Péter Koltai, and Frank Noé. Variational koopman models: slow collective variables and molecular kinetics from short off-equilibrium simulations. *The Journal of chemical physics*, 146(15):154104, 2017.

5.The authors state that they used VMD for trajectory visualization and ChimeraX for AlphaFold model analysis. However, visualization alone does not provide quantitative structural refinement. Given that homology-based models and MD-derived structures can contain local errors, why did not the authors perform explicit structure refinement using loop modeling, Rosetta relax, or MDFF (Molecular Dynamics Flexible Fitting)? Would it not be necessary to ensure that the predicted TMR structures are physically plausible beyond deep learning model outputs?

We respectfully disagree. We used ChimeraX to visualize the confidence scores for each contact provided by the deep learning model. We then compared the pLDDT scores for the TMR in Figure 2 and for the full model in Supplementary Data. To ensure the physical plausibility of the predicted structures, we employed a physics-based method (MD) to minimize, equilibrate, and run simulations for each model. This approach allowed us to refine the structures globally, addressing potential local errors. Given that AlphaFold incorporates energy minimization, we believe MD simulations were sufficient to produce physically plausible structures without requiring additional methods like loop modeling, Rosetta relax, or MDFF.

Results

1.The authors state that their models exhibit significant structural variability, particularly in the arrangement of transmembrane helices. However, they also claim that PCA analysis converges towards two main basins. Given that some methods (e.g., OmegaFold) produce high RMSD values and distinct conformations, while others (e.g., ESMFold) cluster together, how do the authors ensure that this apparent convergence is not an artifact of the simulation constraints rather than a biologically meaningful state? Would longer AA-MD simulations potentially reveal alternative states not captured in the PCA clustering?

We thank the reviewer for this important question.

Deep learning-based structure prediction methods provided us with diverse starting models, exhibiting distinct arrangements of transmembrane helices and contact interfaces (Figure 3). From these models, we generated independent MD trajectories with replicas. Our analyses show that the conformations sampled during the simulations not only recover the predicted contact interfaces from the initial models, but also explore alternative conformations beyond those initial structures.

This indicates that the MD simulations are not constrained to their starting points, but rather allow the system to dynamically explore the accessible conformational landscape, including transitions between different structural basins. This behavior supports the idea that the two main basins observed in

our PCA are not artifacts of the starting models or simulation setup, but represent accessible states .

2.The study compares contact maps across models and finds that some models (e.g., ESMFold, RF, TR) share similar TM2-TM3 interactions, while others (AF2, OF) diverge significantly. However, contact maps only provide static pairwise interactions and do not inherently capture dynamic rearrangements. Given the high flexibility of MCTP4 TMR indicated by MD simulations, would not a time-resolved interaction network (such as correlation-based or dynamic cross-correlation analysis) provide a more robust measure of functionally relevant interactions? How do the authors justify relying on static contact maps when discussing model reliability?

We have performed the cross-correlation analysis on the concatenated trajectory used for the PCA as suggested. The results align with our PCA findings. The analysis reveals that the two helices within each transmembrane hairpin (HP1 and HP2) move as coherent blocks, showing positive correlation within each hairpin. Between HP1 and HP2, we observe a weaker positive correlation, which appears to coincide with the formation of cluster D identified in our PCA analysis. This analysis provides additional validation of the structural dynamics identified in PCA.
This cross-correlation analysis thus provides additional support for the structural dynamics observed in the PCA. These results are included as S17 Figure in the Supporting Information for completeness, though we believe they do not significantly alter the main conclusions drawn from the PCA and clustering analyses.

\textbf{S17 Figure.} Dynamical Cross-Correlation Analysis of TM Helices in the Concatenated Trajectory. The analysis reveals that the two helices within each transmembrane hairpin (HP1 and HP2) move as coherent units, showing strong positive correlation within each hairpin. Between HP1 and HP2, a weaker positive correlation is observed, consistent with the formation of cluster D identified in the PCA analysis.

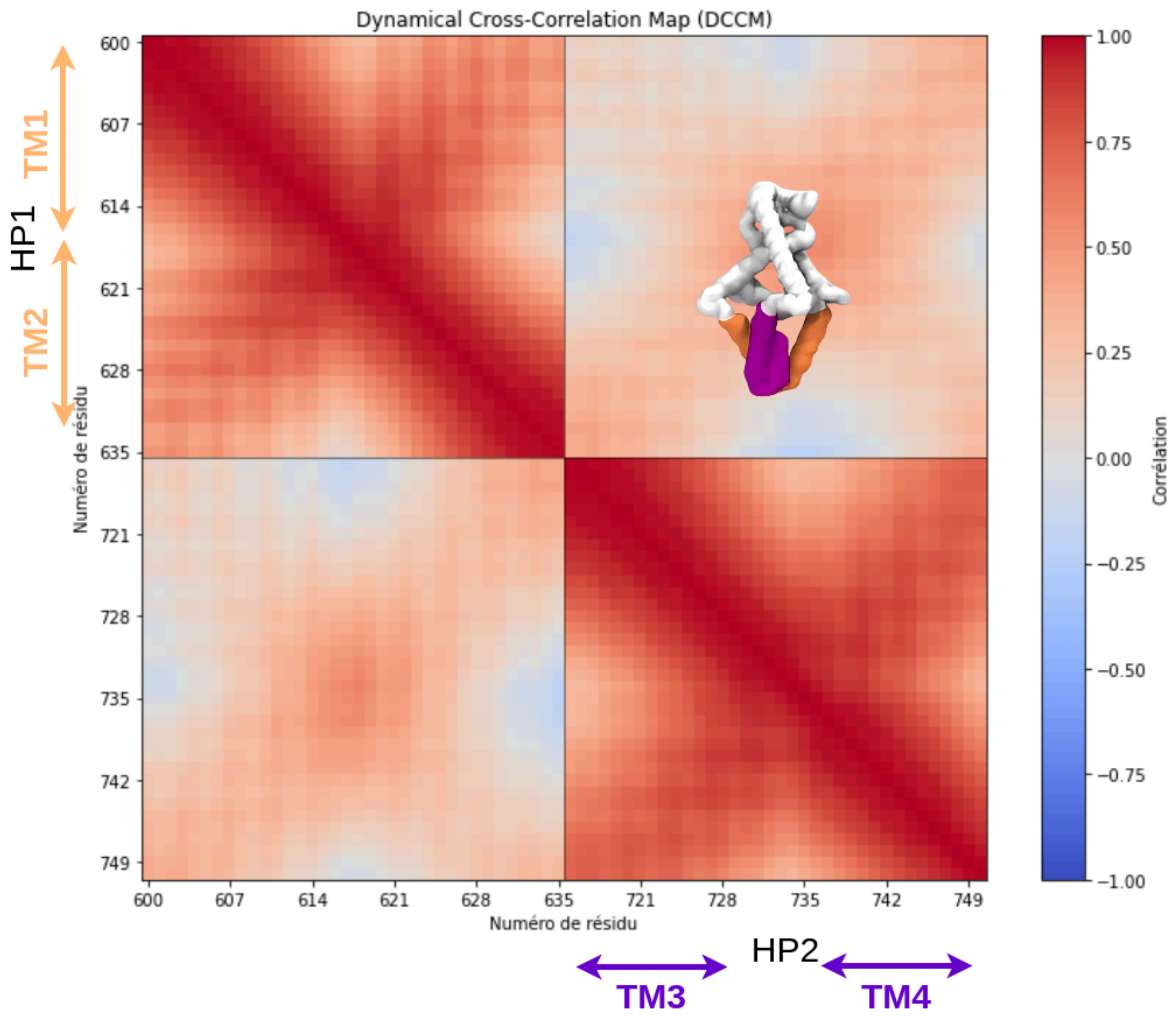

3.The authors removed simulations in which TM domains exited the membrane to ensure consistency. However, excluding these simulations inherently introduces bias - potentially eliminating biologically meaningful states. Would not these deviations indicate that some models are intrinsically unstable within the lipid bilayer, suggesting that the underlying predicted structures might not be valid? Instead of excluding outlier simulations, why not analyze their membrane insertion free energy to determine whether these fluctuations are artifact-driven or biologically relevant?

The transmembrane nature of the HP1–HP2 region is supported by multiple independent tools, including DREAMM, HeliQuest (see additional figure below), and the OPM server (Figure 1), all of which predict this region to be embedded in the membrane. However, when running coarse-grained simulations using the Martini 3 force field, we observed unexpected ejection events, where one or more helices exited the membrane.

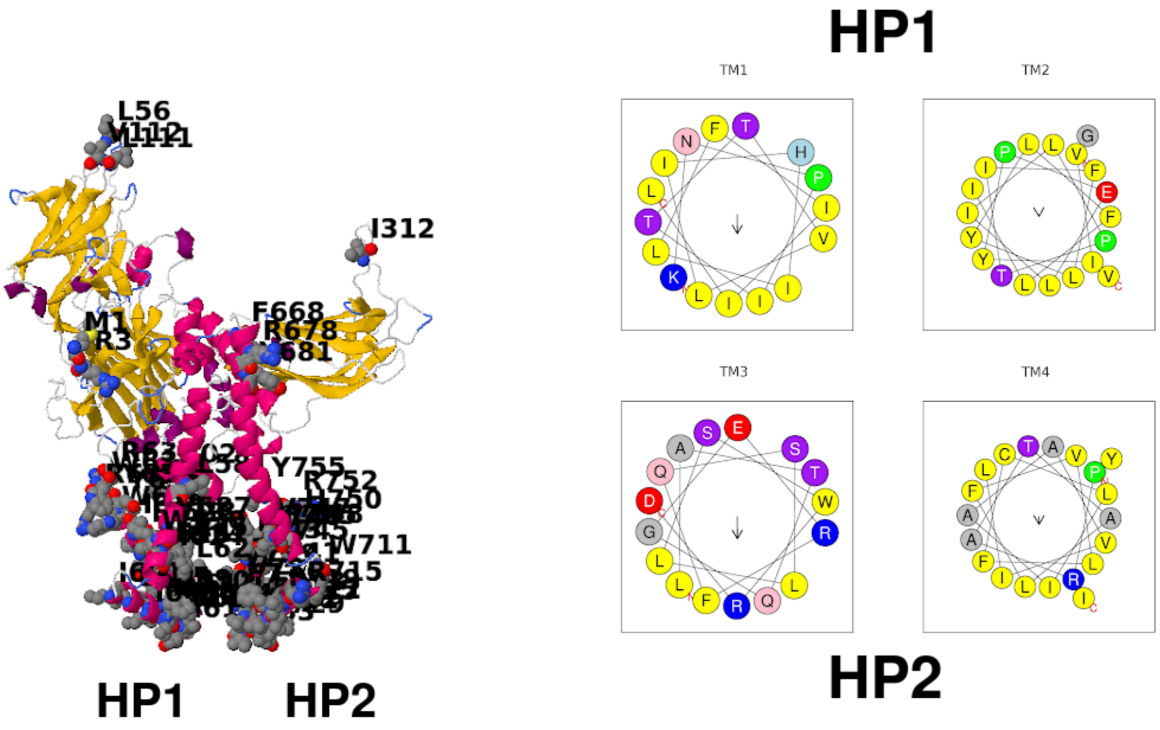

Left: Predicted membrane-interacting regions based on the DREAMM model, visualized as beads. Right: Heliquest prediction for HP1 and HP2 helical. Yellow residues indicate non-polar amino acids, suggesting exposure to the lipid bilayer within transmembrane regions.

Initially, this raised the question of whether such behavior reflected genuine conformational dynamics or instability of the predicted transmembrane segments. To address this, we conducted all-atom simulations of the same systems. In these higher-resolution simulations, the helices remained stably inserted in the membrane, suggesting that the ejections observed in our coarse-grained simulations may not reflect biologically relevant behavior.

Further investigation, supported by recent literature, revealed that Martini 3 may itself be responsible for such artifacts. Specifically, studies have shown that Martini 3 tends to overstabilize protein–water interactions and underestimate the energy barrier for membrane exit, particularly for short or marginally hydrophobic peptides. As a result, artificial ejection events can occur, which are not observed in all-atom simulations or supported by experimental data [7,8].

[7] https://doi.org/10.1016/j.cplett.2023.140436
[8] https://doi.org/10.1016/j.str.2024.08.017

[9]Chatzigoulas, A. and Cournia, Z. (2022). DREAMM: A web-based server for drugging protein-membrane interfaces as a novel workflow for targeted drug design. Bioinformatics, 38(24), 5449-5451.

[10] Gautier R., Douguet D., Antonny B. and Drin G. HELIQUEST: a web server to screen sequences with specific α-helical properties. Bioinformatics. 2008 Sep 15;24(18):2101-2.

4.The AA-MD simulations show that OmegaFold and ESMFold structures remain relatively stable, while AlphaFold2-derived models display higher RMSD values. However, the 250-ns timescale used for these simulations is relatively short, especially for membrane proteins where longer equilibration is often required. Given that coarse-grained models suggest high flexibility and multiple conformational states, how do the authors justify that 250 ns is sufficient to determine stability? Would not longer unbiased simulations or enhanced sampling methods (e.g., milestoning, weighted ensemble, or Markov state modeling) provide a more conclusive assessment of stability and conformational transitions?

We thank the reviewer for this relevant comment.

The main objective of our all-atom MD simulations was not to fully characterize long-timescale conformational transitions, but rather to assess the short-term structural stability of the predicted models, with a specific focus on the behavior of the transmembrane helices. In particular, we aimed to verify whether the helices remained stably inserted in the membrane environment, or whether they tended to drift out which would question their biological relevance.

To further explore the conformational space of each model, we primarily relied on coarse-grained Martini simulations 3µs for each replica, which allow for broader sampling over longer timescales without imposing structural constraints. These simulations were performed in an unbiased manner. Notably, previous studies have shown that helices located outside the membrane core often exhibit amphipathic properties, enabling transient or stable interactions with the membrane surface. Our Martini simulations confirm this behavior and provide insights into alternative membrane-associated conformations that would not be captured by all-atom simulations alone within practical timescales.

5. It appears that several figures are referenced as "??" instead of the correct numbering. This suggests either missing figure captions or an issue with manuscript formatting. This should be carefully revised before submission, as it affects the readability and credibility of the results.

We thank the reviewer for highlighting this issue. The appearance of '??' in figure references was due to a formatting oversight in the initial draft. To address this and ensure consistency with the journal's requirements, we have adopted the official *PLOS ONE* LaTeX template for the revised manuscript, as detailed in our response to the third reviewer's similar comment. All figure references have been carefully checked and corrected to reflect the appropriate numbering, ensuring clarity and readability throughout the document.

To address this problem, we carefully reviewed the manuscript and added \ref{fig} tags for each figure in the LaTeX source file. Wherever appropriate, we also added references to specific panels (e.g., see Fig \ref{fig:5}c) to make the figure citations as clear and precise as possible throughout the text.

Discussion

1.The authors state that four models (ESM, TR, RF, AFM) converged on a similar MCTP4 transmembrane domain, specifically with TM2 and TM3 in close proximity. However, they also note that AF2 and OF produced distinct models and that AF3 showed similarities with OF, while Boltz-1 and Chai-1 aligned with the consensus group. How do the authors justify that the models in the so-called "consensus group" represent the biologically relevant conformation rather than a bias imposed by the prediction algorithms? Could the observed convergence be an artifact of training data or algorithmic assumptions rather than an actual structural feature of the protein?

The "consensus group" (ESMFold, trRosetta, RoseTTAFold, and AlphaFold-Multimer) emerged from our analysis as the models that consistently predicted close interactions between TM2 and TM3. We find other interactions, such as AlphaFold2 and OmegaFold, produced alternative conformations. We provide several lines of evidence supporting the relevance of the consensus conformation:

1. **Convergence during MD simulations:**
   We performed unbiased molecular dynamics simulations starting from all predicted models, including those from OmegaFold and AlphaFold2. Despite their initial differences, these simulations converged toward a conformational basin corresponding to the arrangement predicted by the consensus models, where TM2 and TM3 are in close proximity. This suggests that the TM2–TM3 interaction is a stable and favorable feature of the conformational landscape, rather than an artifact imposed by specific algorithms.

2. **Oligomer prediction analysis:**
   We further predicted oligomeric assemblies of MCTP4. Interestingly, the inter-helical contacts identified in the consensus models were consistently found within intra-chain interactions of the predicted oligomers. In contrast, the alternative interface observed in OmegaFold appeared as an inter-chain contact in these oligomer models. This suggests that the OmegaFold-predicted conformation may reflect a plausible oligomerization interface rather than the native monomeric structure.

3. **Confidence in TM region prediction:**
   AlphaFold2 showed low confidence in the transmembrane region, likely due to the fact that it was trained primarily on monomeric proteins. This limitation can explain the structural divergence and further supports our reliance on methods like AlphaFold-Multimer and ESMFold, which incorporate additional contextual information or training strategies better suited for complex and membrane proteins [10].

[10] Versini R, Sritharan S, Aykac Fas B, Tubiana T, Aimeur SZ, Henri J, Erard M, Nüsse O, Andreani J, Baaden M, Fuchs P, Galochkina T, Chatzigoulas A, Cournia Z, Santuz H, Sacquin-Mora S, Taly A. A Perspective on the Prospective Use of AI in Protein Structure Prediction. J Chem Inf Model. 2024 Jan 8;64(1):26-41. doi: 10.1021/acs.jcim.3c01361. Epub 2023 Dec 20. PMID: 38124369.

2.The authors mention that some TM helices exited the membrane in coarse-grained simulations and that these cases were excluded from further analysis. Given that Martini 3 is known to exaggerate hydrophobic interactions, could the observed membrane exits be a sign of real instability rather than simulation noise ? By removing these cases, is there a risk of overlooking biologically relevant conformations and underestimating the full conformational variability of the MCTP4 transmembrane domain ?
We observed that in a subset of coarse-grained Martini 3 simulations, transmembrane helices were ejected from the membrane. This behavior typically occurred early in the simulation and was irreversible within the simulated timescale.
Given the recent literature on Martini 3 artifacts, we carefully investigated whether these events reflected relevant instability or artifacts of the force field.

Original text :
During our simulations, we investigated the dynamic behaviors of the TM helices of MCTP4 within a lipid bilayer membrane designed to mimic the composition of the endoplasmic reticulum. With Martini 3, we were able to

observe TM Helices that show spatial flexibility. Nevertheless, among the initial 10 replicas for each model, we observed a diverse range of helix behaviors within the membrane. Some helices were coming out of the membrane (Supplementary Material Fig 9-14). Consequently, we repeated the simulations multiple times to obtain a consistent and stable set of results within the membrane. In order to ensure the reliability of our analysis, we excluded simulations in which the TM domains exited the membrane. The Martini 3 force field has been developed in part to solve the issue identified with martini2, that tends to overestimate protein-protein interactions [18].
However, Martini3 is conversely associated with too hydrophobic alpha-helices. Therefore modifications to the scaling of interactions within the Martini 3 force field have been proposed to solve this issue. Thomasen et al., proposed adjusting protein-protein interaction parameters to 88% of their original value in the Martini 3 force field [34]. The authors found that this scaling leads to results that are closer to experimentally observed protein-membrane interactions, compared to a different scaling factor that modifies protein-water interactions. This did not solve the issue in our case (data not shown).

Revised text:
During our simulations, we investigated the dynamic behaviors of the TM helices of MCTP4 within a lipid bilayer membrane designed to mimic the composition of the endoplasmic reticulum. With Martini 3, we were able to observe TM Helices that show spatial flexibility. Nevertheless, among the initial 10 replicas for each model, we observed a diverse range of helix behaviors within the membrane. Some helices were coming out of the membrane (Supplementary Material Fig 9-14). Consequently, we repeated the simulations multiple times to obtain a consistent and stable set of results within the membrane. In order to ensure the reliability of our analysis, we excluded simulations in which the TM domains exited the membrane. The Martini 3 force field has been developed in part to solve the issue identified with martini2, that tends to overestimate protein-protein interactions [18].
However, Martini3 is conversely associated with hydrophilic alpha-helices.
Recent studies have shown that Martini 3 tends to favor adsorbed states over transmembrane (TM) states for short peptides, due to overly strong protein–water interactions. This leads to membrane ejection events that are not observed in higher-resolution simulations. Umbrella sampling confirms that Martini 3 lowers the energy barrier for TM-to-adsorbed transitions and stabilizes the adsorbed state by ~20 kJ/mol. Notably, once a helix exits the membrane, it does not reinsert , consistent with our observations [7]. A similar issue was also reported in recent work [8], where the shorter version of a TM helix (PEPT1-29) exited the membrane, preventing dimerization, while a longer variant (PEPT1-41) showed improved TM stability.

In our case, we observed such exits occurring at different times (early, mid, or late in the trajectory) and across different models, including those initially inserted correctly. To assess whether this instability was a true feature of the protein or a force field artifact, we ran nine independent all-atom simulations (three different models × three replicas each), none of which showed TM helix ejection. This strongly supports the interpretation that the ejections observed in Martini 3 are not meaningful, but rather artifacts of the force field.

We therefore excluded those Martini simulations from our analysis, not to suppress variability, but to avoid drawing conclusions from nonphysical behavior. Importantly, we retained and analyzed several stable Martini simulations (i.e., those in which all TM helices remained embedded), which consistently sampled conformational diversity within the membrane environment, without restraints or bias.

In summary, our exclusion of membrane ejection cases is justified by physical consistency with higher-resolution models and recent literature identifying limitations in Martini 3's treatment of TM peptide stability.

[7] Room for improvement in the initial martini 3 parameterization of peptide interactions.
https://doi.org/10.1016/j.cplett.2023.140436

[8] Versini R, Baaden M, Cavellini L, Cohen MM, Taly A, Fuchs PFJ. Lys716 in the transmembrane domain of yeast mitofusin Fzo1 modulates anchoring and fusion. Structure. 2024 Nov 7;32(11):1997-2012.e7. doi: 10.1016/j.str.2024.08.017.

3.The authors argue that AlphaFold's monomeric predictions may not fully capture evolutionary constraints associated with dimerization, as indicated by low PLDDT scores in TM regions. However, they also state that AFM identifies a strong inter-subunit contact between TM1 and TM4, and that OF predicts a TM1-TM4 contact but considers it an intra-subunit interaction instead. If AFM successfully predicts a dimeric interface, how do the authors conclude that evolutionary constraints are poorly captured? If some models recover these interactions, does this suggest that AlphaFold's handling of multimeric structures is more reliable than implied?

We agree that dimerization constraints could influence AlphaFold predictions. While AlphaFold-Multimer identified inter-subunit TM1-TM4 interactions, OmegaFold treated them as intra-subunit contacts. This discrepancy highlights a potential limitation of AlphaFold's handling of multimeric structures. As we said in our discussion:

Original text

"It is therefore tempting to speculate that evolutionary signals are not necessarily captured by AlphaFold's monomeric models for membrane proteins that form homo-oligomers. In the case of AF2, the model might be subject to conflicting constraints corresponding to intra-subunit and inter-subunit contacts. In agreement with that observation, with AFM the inter-subunit contacts between TM1 from one subunit and TM4 helix from the other subunit are evaluated to be positive see Fig \ref{fig:4}."

Revised text:

It is therefore tempting to speculate that evolutionary signals are not necessarily captured by AlphaFold's monomeric models for membrane proteins that form homo-oligomers. In the case of AF2, the model might be subject to conflicting constraints corresponding to intra-subunit and inter-subunit contacts. We further predicted oligomeric assemblies of MCTP4 to investigate whether the structural features observed in monomeric models were maintained or reorganized upon oligomerization. Notably, the TM2–TM3 contacts identified in the consensus monomeric models (ESM, TR, RF, AFM) were preserved as intra-subunit interactions in the predicted dimers. In addition, AlphaFold-Multimer (dimer and trimer) revealed a simultaneous inter-subunit interface involving TM1 from one subunit and TM4 from the adjacent subunit.

This observation is consistent with the existence of coevolution signals for residues in TM2-TM3 and TM1-TM4, that the prediction tools tend to translate into contacts. Modeling dimers would allow AFM to disentangle those signals and allow it to satisfy both contacts simultaneously. At variance, modeling monomers would favor models trying to satisfy both constraints simultaneously, without success, resulting in a low confidence score.

—---------------------

Reviewer #2: The language particularly in the presentation of the abstract, results and the discussion sections is rather not clear. They are overly informal or imprecise statements. To improve readability, the language should be reworded in a more technical and scientific manner to strengthen the arguments presented in the manuscript and enhance readability.

Abstract

1. On the abstract, consider avoiding general statements and focus on specific results to convey the significance of the study to the reader.

Introduction

Original text

"We found that the TMR of MCTP4 is not rigid, but can adopt various conformations including some not identified by deep learning tools."

Revised text:

"We found that the transmembrane region (TMR) of MCTP4 is not rigid but can adopt multiple conformations. The membrane-embedded region contains two helical pairs: HP1 (TM1–TM2) and HP2 (TM3–TM4). Deep learning predictions revealed three distinct types of inter-helical contact interfaces: ESMFold, AlphaFold-Multimer, trRosetta, and RoseTTAFold consistently predicted a TM2–TM3 contact; AlphaFold2 did not predict any contact between these two helical pairs, while OmegaFold instead suggested a TM1–TM4 interface. Our physics-based coarse-grained simulations not only confirmed the contacts predicted by these models but also revealed a broader conformational landscape. In particular, structural clustering identified five distinct conformational clusters, with additional and more extensive inter-helical contacts not captured by the deep learning predictions."

1. The sentence "These tethering proteins selectively concentrate at plasmodesmata, bind membranes together, and were hypothesised to control the exchange of information between cells" consider rewording the sentence for clarity.

Revised text: "MCTPs localize at plasmodesmata and function as tethering proteins, with their transmembrane region anchored in the endoplasmic reticulum and their C2 domains interacting with the plasma membrane. They are thought to play a crucial role in regulating intercellular signaling"

Methods

1. A cutoff distance of 12 angstrom is used in the contact analysis between the beads "**During the simulations, contacts between the beads representing the helices were defined using a cutoff distance of 12 angstrom, and contact occupancies were calculated for each bead pair**." Please clarify the reason for this threshold.

Revised text: During the simulations, a distance map from 0 to 12 angstrom was used to analyze distance between the membrane-embedded region containing two helical pairs: HP1 (TM1–TM2) and HP2 (TM3–TM4).
This threshold was selected to account for the resolution of coarse-grained Martini models, where inter-residue contact typically span longer distances than in all-atom representations, and to ensure that relevant helix–helix interactions were not missed.

Results
1. Could you rewrite this sentence for clarity, **"This also shows that the system's movement was not ballistic, which suggests that the simulations might have converged to equilibrium."**

Revised text:"**This also indicates that the system did not exhibit ballistic motion, but rather stochastic dynamics consistent with thermal fluctuations, suggesting that the simulations may have reached equilibrium"**

2. What are the metrics for the structural changes when compared to the initial starting points? "In the second cluster (Cluster B), we obtained a representative structure that exhibited significant structural changes compared to the initial starting points. In this structure, helices TM2 are in contact with both TM3 and TM4 helices, which is novel and not captured by deep learning methods (see Figure 5d). This suggests that the simulation explored a different conformational space and underwent substantial structural rearrangements."

Distance maps between HP1 and HP2

3. Could you also include the RMSD values in the statement to justify the comparison in this statement "The simulations started from the model obtained with AlphaFold2 showed higher RMSD in agreement with the observation from coarse grained simulation.

These analyses showed that the RMSD remained below 0.5 nm for the simulations initiated from models obtained with OmegaFold and ESMFold, indicating their relative structural stability. This observation is consistent with the coarse-grained simulations, which also suggested stable behavior for these models. In contrast, the simulations started from the AlphaFold2-derived model exhibited higher RMSD values, reaching approximately 0.8 nm in the all-atom simulations. This increased deviation supports the coarse-grained results and suggests that this conformation is less stable, potentially due to a reduced number of contacts between HP1 and HP2 (see Fig. 3).

Discussion
1. These couple of sentences redundantly emphasize the importance of using

several methods to explore protein conformations “**This also raises the importance of using multiple prediction methods in parallel to explore the conformational landscape of a protein. By relying on a consensus among several methods, we can potentially obtain a more accurate and reliable picture of the protein’s structure. This approach is particularly relevant given the complexity of protein structure prediction and the possibility that different tools have complementary strengths and weaknesses. By combining the results, individual errors can be mitigated, and more robust conclusions can be drawn regarding the likely structure of a protein.**” consider rephrasing for conciseness

Revised text:

These findings highlight the value of using multiple structure prediction methods in parallel to explore a protein’s conformational landscape. Combining predictions leverages the complementary strengths of different tools, reduces the impact of individual errors, and provides a more reliable view of the likely structural states.

2. Could rewrite these two sentences for clarity and conciseness **“This shows how useful it is to use the physics-based MD simulations together with models made by deep learning. By using both methods, we can benefit from their respective strengths and attain a more robust result.”**
Revised text:

This highlights the value of combining physics-based MD simulations with deep learning models, allowing us to leverage their complementary strengths for more robust structural insights.

3. The statement is not clear **“This raises a debate on whether to treat interfaces separately, as suggested by Zhu and colleagues.”**

Revised text:
Predicting the structure of multimers is particularly challenging, as it involves assessing inter-subunit interfaces, which adds complexity compared to monomeric predictions. This has sparked discussion on whether protein interfaces should be modeled independently, as proposed by Zhu and colleagues [44], who suggest that dedicated treatment of interfaces could improve prediction accuracy. More broadly, this raises the question of whether multimeric proteins should be modeled directly as oligomers rather than as isolated monomers, since evolutionary constraints and structural signals may be embedded within their interface regions.

4. Could rewrite these sentences for clarity and conciseness “Overall, our study on

the MCTP4 protein’s transmembrane domain showed that predicting protein structures is complicated and needs a mix of methods. …...We also learned that it’s crucial to think about how proteins group together and that simple models like AlphaFold might not always get it right. This study shows that using multiple approaches is key to understanding protein structures, especially for the parts that are in cell membranes.”

The conclusion has been modified to clarify the main conclusion while preserving conciseness.

5. A dedicated conclusion outlining the implications of the study and key findings could add more value to the work.

Our study demonstrates that the transmembrane region (TMR) of MCTP4 is structurally flexible and capable of adopting multiple conformations. By comparing several state-of-the-art deep learning structure prediction tools, we identified distinct inter-helical contact patterns, with a consensus around TM2–TM3 interactions in several models, and alternative TM1–TM4 contacts in others. Through physics-based coarse-grained simulations, we confirmed the stability of these predicted interfaces and uncovered additional conformational states not captured by static models alone. Structural clustering revealed five distinct conformational clusters, underscoring the dynamic nature of the TMR. These findings highlight the value of combining deep learning and molecular dynamics approaches to better understand the conformational landscape of membrane proteins, particularly for those involved in oligomerization and membrane tethering functions, such as MCTP4.

—------------------

Reviewer #3:

The manuscript by Sritharan et al. presents structural models of the Arabidopsis MCTP4
ER-anchor transmembrane region (TMR) domain using several deep learning-based structure prediction methods. Additionally, the authors performed molecular dynamics (MD) simulations to explore the behavior of the TMR within a lipid bilayer environment. The approach holds promise for capturing the structural complexity and dynamism of the MCTP4 TMR domain within an ER-mimicking bilayer. While the manuscript is well-constructed and the results are interesting, several points need to be addressed before the manuscript can be considered for publication.

Major Comments:

The authors employed the CHARMM36m force field for all-atom (AA) MD simulations. However, the reason behind choosing this particular force field should be clarified, especially in the context of lipid–protein interactions.

CHARMM36m was selected as the force field for all-atom (AA) molecular dynamics simulations due to its established reliability in modeling lipid-protein interactions, particularly within a bilayer environment.

Klauda2010 *The Journal of Physical Chemistry B*, 114. Update of the CHARMM All-Atom Additive Force Field for Lipids: Validation on Six Lipid Types

The authors are encouraged to perform a dynamical cross-correlation analysis to gain insights into the most significant collective motions of the TM helices within the lipid bilayer.

We agree that contact maps alone provide static information. To complement this, we also performed dynamic cross-correlation analysis, as suggested by reviewer 1.

The AA MD simulations are currently limited to 250 ns, which may not be sufficient to fully capture the equilibrium behavior of the system.
Extending the AA MD simulations to at least 1 μs would significantly strengthen the robustness of the conclusions.

We have previously addressed this point in our response to Reviewer 1.

The introduction and discussion would benefit from incorporating recent references

on structure prediction and MD simulations of MCTP4 ER-anchor TMR domains, as well as a clearer articulation of how this study advances the current understanding of MCTP4 structure and function.

We thank the reviewer for this valuable suggestion and agree that updating the introduction and discussion with recent advances strengthens the manuscript. Accordingly, we have incorporated references to the latest generation of structure prediction tools, including AlphaFold 3, Chai-1, and Boltz-1, which are trained on expanded datasets and include a broader range of molecular types, enhancing their predictive accuracy for complex proteins like MCTP4 (see figure below). Additionally, we have added a reference to a recent study employing Martini coarse-grained molecular dynamics (MD) simulations, which demonstrated that the C2 domains of MCTP4 interact with PI4P lipids [11], providing insights into their membrane-binding behavior. We also included a reference to the work of Patrick Li et al., which highlights the critical role of MCTP4 in stabilizing endoplasmic reticulum (ER) connections across plasmodesmata [12]. Their findings show that mutations in the transmembrane (TMR) domain destabilize these channels, leading to their complete closure, underscoring the functional importance of the TMR .

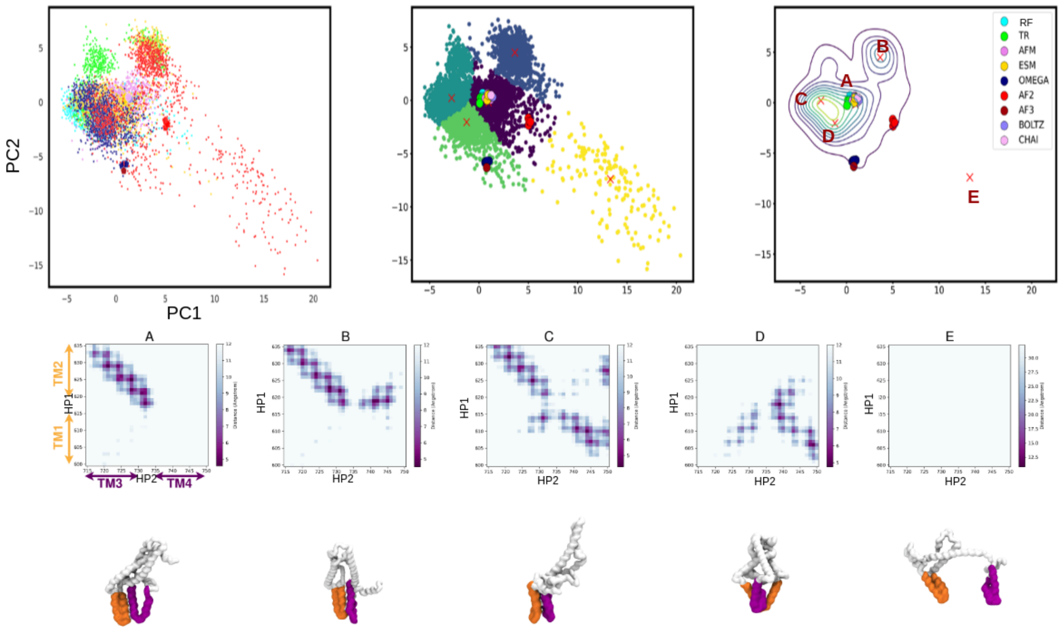

By characterizing the collective motions and stability of the TM helices within the lipid bilayer, our study elucidates the dynamic interplay between the TM and C2 domains, offering a more comprehensive view of MCTP4's role in ER-plasmodesmata connectivity.

[11]http://dx.doi.org/10.2139/ssrn.4682263
[12]DOI: 10.1126/science.adn4630

Minor Comments:
The figure numbers are missing in several places within the main text, making it difficult to follow the results.

We thank the reviewer for pointing out this issue. To ensure consistency with the journal's formatting requirements, we chose to use the official PLOS ONE LaTeX template. We have previously addressed this point in our response to Reviewer 1.

The "Jupyter Notebook" section in the Materials and Methods should be removed, and the corresponding GitHub link can be provided under the Data Availability section instead. Typos and grammatical issues should be carefully checked

The "Jupyter Notebook" section has been removed from Materials and Methods, and the corresponding GitHub link has been relocated to the Data Availability section.

Revised text :
"2.8 Data analysis and visualisation
All of the analysis and data used in this study have been documented and made available for reference and reuse. These scripts are hosted in Jupyter Notebooks, a popular open-source web application that allows for the creation and sharing of documents. The notebooks can be accessed from the associated GitHub repository at https://github.com/Jouffluu/Molecular-modelling-MCTP.
Some basic analysis tasks, such as the calculation of the root mean square deviation (RMSD) or the distances between the centres of the geometry of the two helices, were performed using the tools provided in the GROMACS 2021.5 software package."
